# Roles of dimeric intermediates in RNA-catalyzed rolling circle synthesis

**DOI:** 10.1093/nar/gkaf057

**Published:** 2025-06-16

**Authors:** Emil L Kristoffersen, Ewan K S McRae, Niels R Sørensen, Philipp Holliger, Ebbe S Andersen

**Affiliations:** Interdisciplinary Nanoscience Center, Aarhus University, 8000 Aarhus, Denmark; MRC Laboratory of Molecular Biology, Francis Crick Avenue, Cambridge CB2 0QH, United Kingdom; Interdisciplinary Nanoscience Center, Aarhus University, 8000 Aarhus, Denmark; Center for RNA Therapeutics, Houston Methodist Research Institute, Houston, TX, 77030, USA; Interdisciplinary Nanoscience Center, Aarhus University, 8000 Aarhus, Denmark; MRC Laboratory of Molecular Biology, Francis Crick Avenue, Cambridge CB2 0QH, United Kingdom; Interdisciplinary Nanoscience Center, Aarhus University, 8000 Aarhus, Denmark

## Abstract

The RNA world hypothesis is supported by the discovery of RNA polymerase ribozymes that can perform RNA-catalyzed RNA replication processes on different RNA templates. Recently, RNA-catalyzed rolling circle synthesis (RCS) on small circular RNA (scRNA) templates has been demonstrated. However, the structural and dynamic properties of scRNA replication and its products and intermediates have not been explored. Here, we have used cryogenic electron microscopy (cryo-EM) to characterize products and intermediates relevant for RCS replication. We find that these form an unexpectedly diverse group of RNA nanostructures. The main structural motif observed is a fully hybridized dimeric complex composed of two scRNAs and their complement strands resolved to 5.3 Å. Cryo-EM also reveals higher-order dimer filaments and dimer assembly intermediates, suggesting an assembly mechanism for the observed complexes. We show that the dimer complexes are stable and inhibit RNA-catalyzed RCS but can be reactivated by addition of more scRNA templates. We propose dimer formation as a general property of RCS replication and speculate that dimers might have benefited a primordial RNA genetic system by providing a stable ‘‘storage’’ form for RNA replication products and by coordinated RNA replication on both scRNA template strands.

## Graphical Abstract

**Graphical Abstract:**
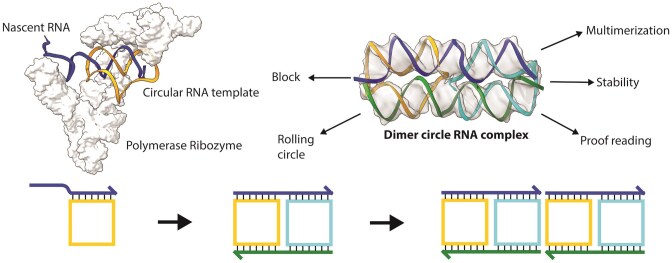

## Introduction

How life and its first genetic system may have emerged on the early Earth is an area of active investigation. Among various competing ideas, the so-called RNA world hypothesis [1, 2] remains an attractive proposition due to its conceptual simplicity and compelling supporting evidence in modern biology. This includes the role of RNA catalysts (ribozymes) in universal biological processes such as translation and RNA processing, and the presence of ribonucleotide cofactors in central metabolic processes. The RNA world is further supported by the demonstration of plausible prebiotic chemistry routes toward the synthesis of activated ribonucleotides [3–7] and the nonenzymatic, templated polymerization of RNA [8–11].

While a cornerstone of the RNA world hypothesis—sustained RNA-catalyzed RNA self-replication—remains to be demonstrated, *in vitro* evolution has enabled the discovery of RNA polymerase ribozymes with increasing synthetic power [12], including the synthesis of active ribozymes [13, 14], longer-than-self RNA products on some templates [15], and processive polymerization and promotor recognition [16]. Recently, a polymerase ribozyme, which utilizes trinucleotide triphosphates (pppNNN, triplets) rather than ribonucleotide triphosphates (NTPs) as substrates, has been described [17]. This triplet polymerase ribozyme (TPR) is a heterodimer composed of a catalytic subunit (5TU) and an inactive accessory subunit (t1) as confirmed by its cryogenic electron microscopy (cryo-EM) structure [18]. This TPR is able to copy highly structured templates as well as segments of itself [17].

While there is significant progress in obtaining increasingly active polymerase ribozymes, sustained RNA replication will likely also require strategies for strand separation as duplex RNAs are exceedingly stable. Among different, conceivable strategies for strand separation, rolling circle synthesis (RCS) is an attractive mode as it mimics the simple replication strategies of viroids (and some viruses) in biology, which have themselves been suggested to predate cellular life [19] and be relics from the RNA world [20]. However, these natural replicons use protein-based polymerases to replicate, which were likely not present in an RNA world.

An important feature of RCS is that the energy released from the polymerization reaction can be directed to drive strand displacement [21, 22] and result in cotranscriptional displacement and folding of the nascent strand. Therefore, RCS potentially enables the linkage of genotype (RNA template) with the RNA phenotype (folded nascent strand)—a prerequisite for evolution—without compartmentalization. In contrast, polymerization on linear strands leads to the formation of highly stable RNA duplexes that require an external energy input to dissociate duplex strands for continued replication. This has previously been explored using temperature cycling, wet–dry cycling, or pH cycling [23, 24]. While RCS requires the formation of circular RNA (circRNA) templates, these have been shown to arise from self-ligation processes [25, 26] or wet–dry cycling [27, 28], suggesting multiple prebiotic routes to circRNAs.

Recently, we reported that the TPR can perform RCS on small circular (sc)RNA templates [29]. The RCS was made possible because hybridization of small RNA circular templates and their growing nascent strand is energetically disfavored, provided the scRNA is considerably smaller than the RNA double helix persistence length of ∼300 bp [30]. Indeed, molecular dynamics (MD) simulation showed that a circular template of 36 nt could maximally form a 24-bp duplex by hybridizing with its complementary linear strand. Upon further extension (beyond 24 nt), the MD simulation suggested that the system is dynamic, leading to unpaired 5′- and 3′-ends of the nascent strand and unpaired segments in the scRNA template, which we proposed would facilitate displacement of the 5′-end from the template upon iterative extension of the 3′-end. However, while the TPR was able to synthesize nearly three rounds of replication on the scRNA [29], we observed a strong inhibition of synthesis after completion of just one round on the scRNA template, which was hard to rationalize based on our MD model.

To gain deeper insight into the RCS process and the strong inhibition observed upon full-length circle synthesis, we used cryo-EM to study the structure of putative RCS replication products formed by complexing the full-length scRNA and nascent complementary RNA (cmpRNA). To our surprise, cryo-EM revealed not one by a diverse variety of distinct structural species identified as monomers, dimers, and multimers of the scRNA–cmpRNA complex. The main structural complex, which was resolved to an overall resolution of 5.3 Å, was composed of two scRNAs and two cmpRNAs forming two interconnected parallel RNA helices. This dimer structure was further investigated in primer extension assays confirming it as an inhibitory product that could, however, be reactivated by the addition of an excess of scRNAs.

## Materials and methods

### RNA preparation

RNA was prepared essentially as described in [29]. In brief, RNA was *in vitro* transcribed or chemically synthesized (IDT). When relevant, polynucleotide kinase (NEB) was used to remove 2′-3′ cyclic phosphate. scRNA was prepared by T4 RNA ligase 1 ligation (NEB) exactly as described in [29]. RNA was then gel purified by 20% (denaturing) urea polyacrylamide gel electrophoresis (PAGE), detected by UV shadowing, and recovered by freeze and squeeze extraction [removing gel pieces with a Spin-X column (0.22 μm pore size, Costar)]. When relevant, cmpRNA (500 pmol) was 5′-end ^32^P ‘hot’ labeled using fresh γ-^32^P-labeled ATP (50 nmol) and PNK (NEB) following the producer’s recommendations. Excess γ-^32^P-labeled ATP was removed during PAGE purification. Fluorophore-labeled cmpRNA was commercially acquired (IDT). Eventually, this procedure led to gel-purified circularized scRNA and gel-purified cmpRNA (in some cases labeled with radioactive 5′-phosphate or FAM fluorophore).

### RNA circularization

Gel-purified linear scRNAs were circularized as described in [29]. In short, 5′-phosphorylated and 3′-OH RNA was circularized with RNA ligase 1 (NEB) followed by denaturing PAGE purification as described earlier. The gel band representing circularized RNA monomer (scRNA) was identified as described in [29].

### Assembly of the cmpRNA and scRNA structures and PAGE analysis

Gel-purified cmpRNA (with or without label as specified in the text) and scRNA were mixed in the reaction buffer (50 mM Tris, pH 8, 100 mM MgCl_2_) at noted molar ratios and incubated for 10 min at room temperature and then adjusted to reaction conditions. After this, the samples were stored on ice for up to a few hours until used. Assembled RNAs, including relevant control samples, were analyzed by denaturing (8 M urea) PAGE (10% or 20% as noted in the text) running in 1× TBE buffer at 25 W for 1–2 h or by (native) PAGE where the gel was cast with 1× TBE and 100 mM MgCl_2_ and run at 100 V for 16 h. Gel-separated radioactive RNA was detected using radioactive films (GE HealthCare) and scanned using a Typhoon scanner (Thermo Scientific). FAM fluorophore-labeled RNA was analyzed directly in the gel by a Typhoon scanner.

### Cryo-EM

RNA for cryo-EM was prepared as described earlier by mixing equimolar amounts of scRNA and cmpRNA, followed by precipitation in 96% EtOH + KCl and washing in 70% ice-cold EtOH, and was finally redissolved in buffer (50 mM Tris, pH 8, 100 mM MgCl_2_) to a final concentration of ∼6 mg/ml. All buffers and EtOH solutions were filtered (3 K cutoff, Amicon) prior to use. Finally, the RNA was added to grids for downstream cryo-EM analysis as described below.

### Cryo-EM data acquisition

Protochips 1.2/1.3 300 mesh Au-Flat grids were glow discharged in a GloQube Plus glow discharging system for 45 s at 15 mA and used immediately after for plunge freezing. Plunge freezing was performed on a Leica GP2 with the sample chamber set to 99% humidity and 15°C. Three microlitres of sample was applied onto the foil side of the grid in the sample chamber before a 4 s delay and then 6 s of distance-calibrated foil-side blotting against a double layer of Whatman #1 filter paper. With no delay after blotting the sample was plunged into liquid ethane set to −184°C. All data were acquired at 300 keV on a Titan Krios G3i (Thermo Fisher Scientific) equipped with a K3 camera (Gatan/Ametek) and energy filter operated in EFTEM mode using a slit width of 20 eV. Data were collected over a defocus range of −0.8 to −2 micrometres with a targeted dose of 60 electrons per square Å^2^. Automated data collection was performed with EPU, and the data were saved as gain normalized compressed tiff files with a calibrated pixel size of 0.647 Å per pixel.

### Single particle image processing and 3D reconstruction

A total of 7406 movies were recorded and pre-processed with cryo-EM single particle *ab initio* reconstruction and classification (cryoSPARC) live (CS-live), which performed motion correction, contrast transfer function (CTF) correction, micrograph curation, and initial particle picking. Manual processing was continued using 2D classes from the automated live processing, which was set up to use blob picking with a 220 Å blob diameter, extract particles with 512 px box size, and bin to 128 px. From these initial 2D classes, it was apparent that some of the particles were small, and some were large, extending out of the 330 Å box (Supplementary Fig. S2).

Three 2D classes that represent the smaller particles (Supplementary Fig. S3A) were used as templates for templated particle picking with a particle diameter of 150 Å. From this, 668 921 particles were extracted with a box size of 512 px and binned to 128 px. Three-class *ab initio* reconstruction using 60 000 randomly selected particles followed by heterogeneous refinement using the entire particle stack was performed (Supplementary Fig. S3B). This generated one junk class, one class resembling a single helix (class 1), and one class resembling two adjacent helices (class 3).

*Class 2*: Class 2 and class 3 particles were derived from the 243 227-particle stack in Supplementary Fig. S3B. A five-class *ab initio* reconstruction followed by heterogeneous refinement split the particles into one class containing the lariat-like class 2 particles, two classes containing class 3 particles, and two classes that were discarded as junk (Supplementary Fig. S3C). The 48 761 class 2 particles were further aligned by homogeneous refinement followed by local refinement with default parameters. The final gold-standard Fourier shell correlation (GSFSC) analysis fell below 0.143 at 7.8 Å and local resolution analysis showed that the majority of the map is between 7 and 8.5 Å resolution; once again, a viewing angle distribution plot indicates considerable preferred particle orientation.

*Class 3*: The red boxed two classes from Supplementary Fig. S3F were combined and re-extracted with a box size of 432 px binned to 216 px. These 117 663 particles were further aligned with homogeneous refinement followed by local refinement using a pose and shift Gaussian prior and minimizing over per-particle scale. This achieved a GSFSC of 6.3 Å at 0.143; aligning to the symmetry axis and enforcing C2 symmetry improved the numerical resolution to 5.17 Å but made negligible difference to the map.

*Class 1*: The particles comprising class 1 were deemed too noisy for effective sorting only in 3D and so the 668 921-particle stack was classified in 2D (Supplementary Fig. S3D) and only the ‘‘shinier’’ looking classes (Supplementary Fig. S2) were used for further processing of class 1. These 436 781 ‘‘shiny’’ particles were used in three-class *ab initio* using 60 000 randomly selected particles followed by heterogeneous refinement using the entire particle stack (Supplementary Fig. S3E). While this cleaning of the particle stack by 2D sorting seems to have improved the resulting 3D reconstructions for class 1-like particles, when processing was continued for the class 3-like particles from this ‘‘cleaned’’ stack, we attained lower resolution final reconstructions than when no sorting in 2D was performed.

The 114 396 class 1-like particles were further sorted in 3D by a two-class *ab initio* reconstruction using 30 000 random particles followed by heterogeneous refinement using the full particle stack (Supplementary Fig. S3F). This resulted in 66 279 particles sorting into a class 2-like lariat and 48 117 particles into what we refer to as class 1, the smallest sized reconstruction approximating a single double helix with a moderate protrusion. Nonuniform refinement gave best looking unsharpened map, which we used for structural analysis of class 1. While the GSFSC resolution at 0.143 cutoff is 7.29 Å as measured in cryoSPARC, local resolution analysis shows that the majority of the map is more comfortably resolved between 8 and 9 Å. These particles have considerable orientational bias, limiting the resolution that can be obtained and introducing anisotropy in the map.

*Classes 4 and 5*: For the larger classes, we first re-extracted the particles from the 2D classes from the CS-live blob picking that were elongated (Supplementary Fig. S4A), this time using a box size of 1024 px binned to 256 px. Two-class *ab initio* reconstruction with these 33 377 particles yielded one longer and one intermediate size map (Supplementary Fig. S4B). Particles from the longer class were used for 2D classification into 20 classes, 3 of which were used for templated particle picking (Supplementary Fig. S4C). A total of 202 978 particles were extracted with a box size of 1024 px binned to 256 px. To isolate the longer filaments from smaller particles, we used three-class heterogeneous refinement using the long class from the previous *ab initio* model as one input volume, class 3 map as another volume, and a small junk volume as a third volume (Supplementary Fig. S4D). This resulted in 130 660 particles being sorted into the longer filament class (Supplementary Fig. S4E).

From here, we started a three-class *ab initio* reconstruction and heterogeneous refinement as well as a five-class *ab initio* and heterogeneous refinement. The intermediate length filament from the five-class 3D sorting yielded the best-resolved dimer of dimer map (class 4) after local refinement reaching 9.3 Å with 26 859 particles (Supplementary Fig. S4F). The longer class (50 298 particles) from the three-class 3D sorting (Supplementary Fig. S4G) was further split by an additional round of three-class *ab initio* into heterogeneous refinement (Supplementary Fig. S4H), yielding three similar reconstructions, one of which, upon local refinement, displayed more helical features than the others (class 5: 18 173 particles, 9.6 Å). Because of the split processing streams having the potential to contain the same particles in the class 4 and class 5 reconstructions, we intersected the particle stacks to remove any overlap, of which there were some 2000 particles.

Parameters for global and local resolution estimation as well as particle orientation for the cryo-EM density maps of classes 1–5 are shown in Supplementary Figs S5–S9.

### Model building

From the reconstructed volume of the medium-sized particles, it was clear that the length was the correct size for a fully hybridized 36-nt component and that the full volume must be comprised of two circular and two linear components. Due to the repetitive nature of the sequences, there were multiple possibilities for the hybridization of two circles with two linear complements. Different lengths of double-stranded RNA were generated using RNAbuild [31] and fit into volume to determine how many base pairs were present on each half of the circular components before the crossover. It was determined that the optimal solution was to have the short side with 14 bp and the longer side with 22 bp.

Model building was performed in UCSF ChimeraX version 1.3 [32–35] using ISOLDE to perform MD with flexible fitting with a low (500 kJ mol^−1^ (map units)^−1^ Å^3^) weight on the map and 0 as the temperature factor [32, 34, 35]. The pdb was sequence-corrected and each chain renumbered using PDB-Tools [36]. The model was then refined in real space using the Phenix software package to optimize bond angles and then relaxed in ISOLDE MD force field (MDFF) to reduce the clash score before a final validation using the Phenix software package [37–41].

### Ribozyme (TPR)-catalyzed polymerization of cmpRNA

To investigate whether dimer could work as a template for TPR, scRNA and cmpRNA were mixed and incubated as described earlier. After incubation, dimer was either purified or directly used for ribozyme polymerization assay.

**Figure 1.**
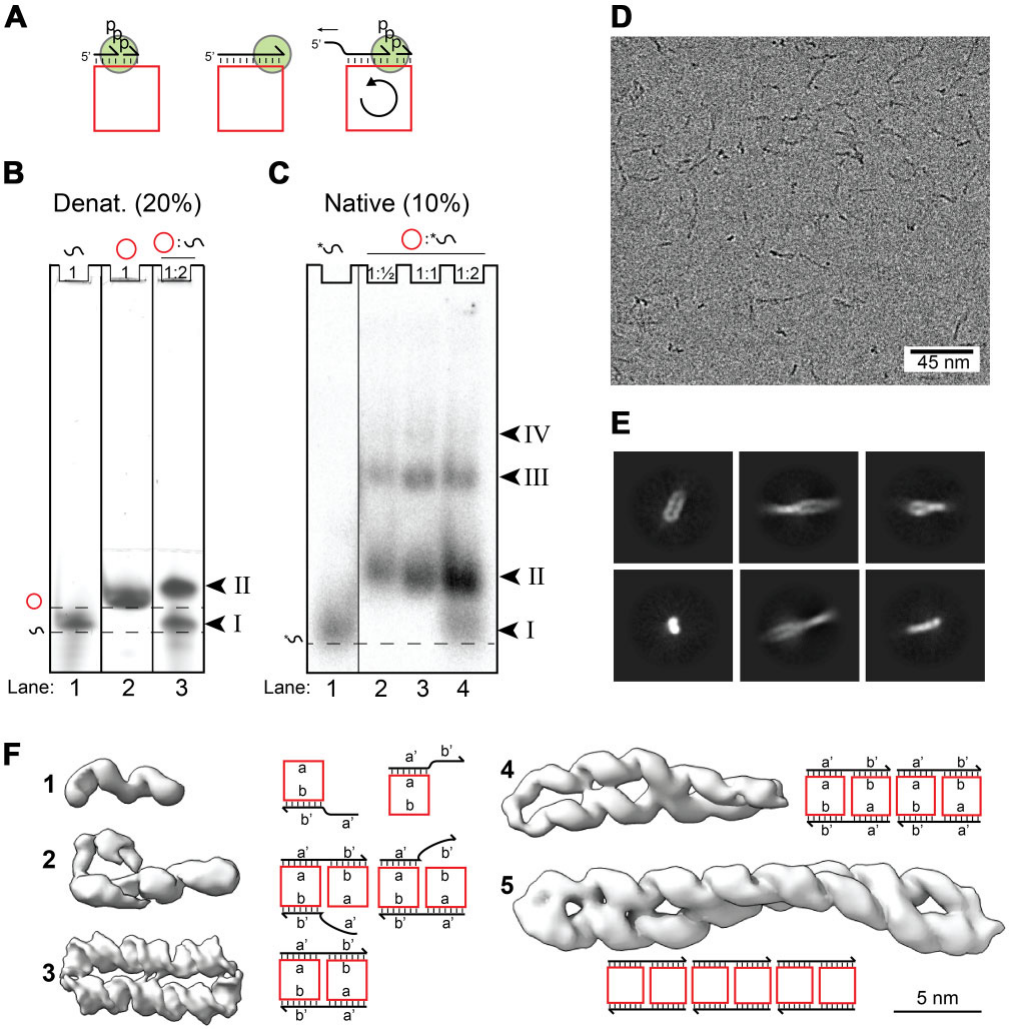
Complexes between circular template and linear product strands. (**A**) Schematics showing the basics of RCS, where the scRNA (square) binds a primer (one-sided arrow) that is elongated at the 3′-end by an RNA polymerase ribozyme (circle). When RCS reaches a certain length, the 5′-end is released from the template due to strain in the scRNA. (**B**) 8 M Urea denaturing (20%, 19:1) polyacrylamide gel with RNA constructs. RNA was stained with SybrGold. Arrowheads and Roman numbers denote bands in the gel. The dashed line represents the front of band migration of selected species. (**C**) Native (10%, 37:1) polyacrylamide gel. Bands observed in the native gel represent 5′-end radio labeled cmpRNA. Whole raw images of gels shown in panels (B) and (C) are provided in Supplementary Fig. S1A and B, respectively. (**D**) A representative cryo-EM micrograph. (**E**) Selected 2D classes. (**F**) The five structural classes observed by cryo-EM and their interpretations as monomer, dimer, and multimer species. Domains of the circle are denoted by a′ and b′ and the complementary domains on the product strand are denoted a’ and b’.

For gel purification of dimer, hot-labeled cmpRNA and scRNA were prepared as described earlier and gel extracted by native PAGE. All three visible bands (bands I–III in Fig. 1B) were dissected from the gel and the RNA extracted while keeping the RNA at nondenaturing conditions at all times. Gel-purified native RNA (normalized according to radioactive counts) was then incubated with or without active TPR (5TU/t1) (5 pmol) and triplets [pppGAA and pppCUG (100 and 50 pmol, respectively)] in the reaction buffer (10 μl total volume) and frozen on dry ice followed by incubation at −7°C for 7 days. Some samples were diluted by adding 490 μl H_2_O prior to freezing. As a positive control, TPR and triplets were mixed with a linear template (same sequence as scRNA) and a fluorophore-labeled primer (template: D; primer: P9 [29]) using the same reaction mix and reaction buffer.

For primer-extension experiments, fluorophore-labeled cmpRNA (5 pmol) and scRNA (5 pmol) were prepared as described earlier to assemble the dimer. After dimer assembly, active TPR (5TU/t1) (5 pmol), triplets [pppGAA (100 pmol), pppCUG (50 pmol), pppGAC (50 pmol), pppAUC (50 pmol), and pppCGG (50 pmol)], and reaction buffer components were added (final volume of 10 μl in reaction buffer). For some samples (noted in the text), additional scRNA (5 pmol) was added to the preassembled dimer and immediately before the addition of the TPR and the rest of the reactants. Finally, all samples were frozen on dry ice followed by incubation at −7°C.

After incubation at −7°C for 7 days, samples were thawed, adjusted to 500 μl with H_2_O, and RNA was precipitated by addition of EtOH + KCl (using glycogen carrier) to 70%. Precipitated RNA was redissolved in loading buffer (95% formamide, 25 mM EDTA, and bromphemol blue) and mixed with 1 μM nonlabeled cmpRNA to ensure separation of the labeled constructs from the templates. Note that this is important to release the labeled cmpRNA from the very stable dimer complex and allow detailed PAGE analysis. Finally, the samples were analyzed by denaturing PAGE. Radioactive RNA was detected using radioactive films (Thermo Scientific) and scanned in a Typhoon scanner (Thermo Scientific) using phosphorescence mode. Fluorophore-labeled RNA was scanned directly in the Typhoon scanner using fluorescence mode.

### RNA hydrolysis assay

To compare RNA stability, equal amounts of gel-purified dimer (band II) or single-stranded cmpRNA (band I) (measured by radioactivity) were prepared in reaction buffer and incubated at −7°C for 7 days. Following incubation, samples were investigated by denaturing PAGE and radioactivity detection as described earlier. Fraction of hydrolysis (per 7 days at −7°C) was calculated as the signal below the full-length cmpRNA band divided by the total signal of the lane. The calculated fraction of hydrolysis was plotted in a bar chart using Excel (Microsoft).

## Results and discussion

### Cryo-EM structures of the full-length RCS product

We previously demonstrated RNA-catalyzed RCS through primer extension on an scRNA template by a TPR (Fig. 1A) [29]. In this study, we detected a strong inhibition of the RCS reaction at one round of synthesis, i.e. full-length synthesis on the scRNA template, an observation that was hard to rationalize from our limited model of the RCS replication [29]. To explore the molecular nature of this inhibitory state, we studied the hybridization between a full-length 36-nt cmpRNA (equivalent to a full-length RCS synthesis product) and a 36-nt scRNA template without the presence of TPR. Denaturing gel electrophoresis showed that cmpRNA and scRNA have different individual electrophoretic mobilities (Fig. 1B, lanes 1 and 2). However, when hybridized, they form a distinct lower mobility band (Fig. 1B, band II in lane 3), which is stable under denaturing conditions, suggesting the formation of a highly stable complex. Next, we performed native gel electrophoresis to verify the formation of a complex between circular and linear RNAs, using radiolabeled cmpRNA and unlabeled scRNA (Fig. 1C). In addition to a major band (Fig. 1C, band II) that may correspond to band II in Fig. 1B, we observed two slower migrating bands (bands III and IV) that are not present in Fig. 1B and may therefore represent weaker interacting multimers of band II.

Next, a sample with equimolar amounts of scRNA and cmpRNA was analyzed by cryo-EM. The micrographs and class averages revealed both distinct particles and longer filaments (Fig. 1D and Supplementary Fig. S2). Using an iterative 3D reconstruction approach, we identified five distinct structural classes (Fig. 1E, Supplementary Figs. S2–S9, and Supplementary Table S2; for details including local resolution ranges, see the ‘‘Materials and methods’’ section; resolutions mentioned in the main text is GSFSC 0.143). All classes showed clear helical features, providing confidence in identifying them as genuine RNA complexes. Class 1 showed a density corresponding to two helical turns of an RNA double helix. Class 2 showed density similar to two parallel RNA helices, one long and one shorter. Class 3 led to our best-resolved structure showing two parallel helixes, each having slightly >3 helical turns. We presume that this structure corresponds to band II in the denaturing gel in Fig. 1B as this structure is likely highly stable. Finally, classes 4 and 5 displayed long twisted structures approximately double and triple the length of class 3, and likely correspond to bands III and IV in the native gel in Fig. 1C.

Based on the identified structures, we concluded that class 3 likely represents a homodimer between two scRNA–cmpRNA complexes that have dimerized by strand exchange and mutual cross-hybridization. This process is likely driven by the geometrical restriction of the small circular template and the rigidity of the RNA duplex, which makes hybridization of the cmpRNA to the scRNA energetically unfavorable beyond 24 bp as found by MD simulation [29]. Classes 4 and 5 represent multimers formed by end-to-end stacking of the class 3 dimers. Classes 1 and 2 were poorly resolved but resemble RCS structures as predicted by our MD simulation [29] and may represent intermediates trapped on the way to dimer formation. Furthermore, class 2 has the potential to form via two different pathways where either the 5′- or 3′-end does not anneal. These interpretations are schematically illustrated next to the density maps in Fig. 1F. Next, we examined the individual structural classes in more detail, starting with class 3.

### Structure of fully hybridized dimer

**Figure 2.**
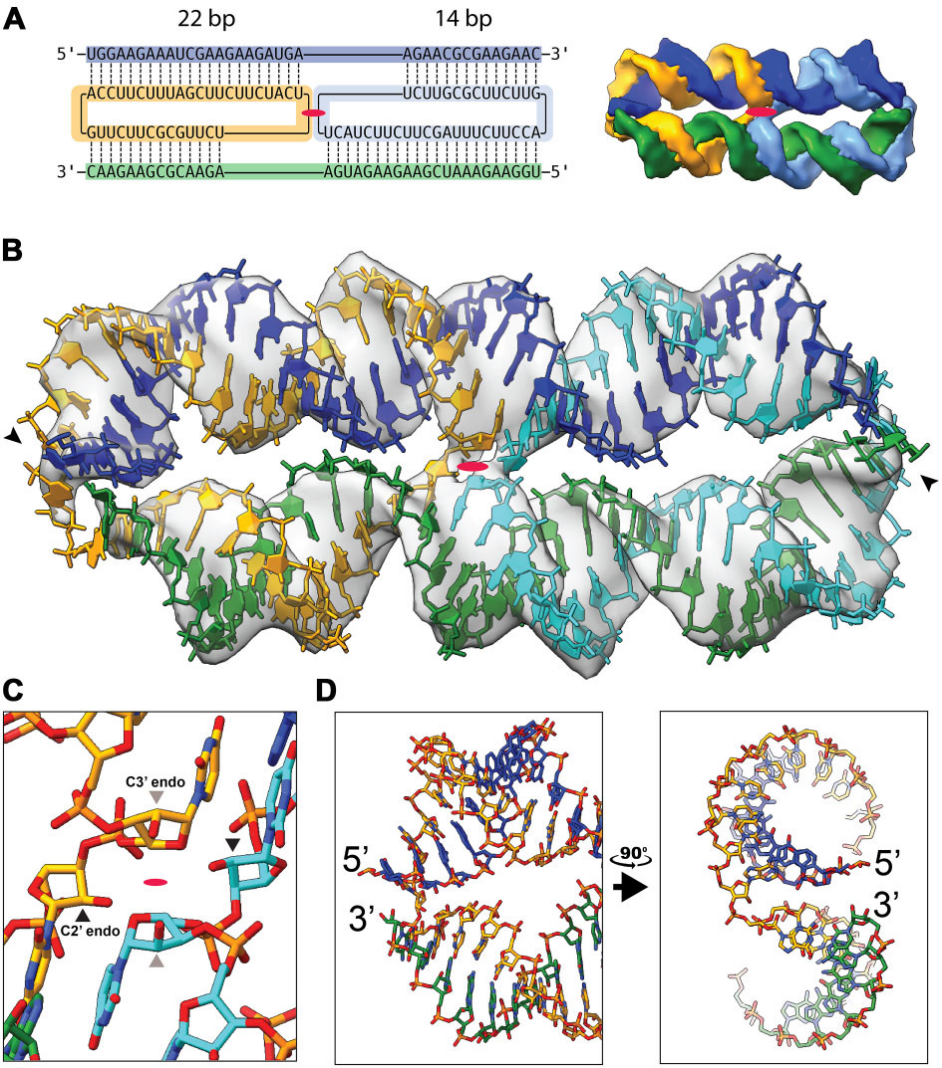
Structure of the fully hybridized dimeric circle–product complex (class 3). (**A**) Secondary and tertiary structure model of two scRNAs bound to their product strands with domains of 22 and 14 bp as suggested from the cryo-EM data. Ellipse indicates C2 symmetry axis. (**B**) Atomic model based on the density of cryo-EM class 3. Arrows indicate 5′-end. Ellipse indicates C2 symmetry axis (EMDB: EMD-19759; PDB: 8S6W). (**C**) Zoom on the crossover junction where the 5′ nucleotides entering the junctions are found in the C2′-*endo* conformation. (**D**) Zoom on helix ends where the scRNA crossover from one helix to the other is shown in two perpendicular views.

The dimer from class 3 was found to be composed of two scRNAs and two cmpRNAs that are fully hybridized (Fig. 2A). The cryo-EM reconstruction using C2 symmetry led to our highest resolution map of 5.3 Å. Based on this map, we built an atomic model, which fitted the density well consisting of two parallel RNA helices of 36 bp connected by anti-parallel crossovers formed by the two scRNAs—a central two-strand crossover and two proximal one-strand crossovers (Fig. 2B; see Supplementary Fig. S7 for comparing C1 and C2 symmetries). A nonideal crossover spacing is observed, with 22 bp on one helix and 14 bp on the other. The 22-bp segment has two helical turns between the crossovers (corresponding to an ideal spacing of 11 bp per turn), whereas the 14-bp segment only has one turn. The nonideal crossover length of the 14-bp segment results in its underwinding and a global distortion of the structure where the 22-bp helix bends outward and the 14-bp segment bends inward giving the structure a slightly curved shape (Fig. 2B).

The model suggests that the central crossover in the dimer forms a C2′-*endo* conformation adopted by the nucleotide at the 5′-end of the strand entering the crossover (Fig. 2C). This conformation was introduced by the refinement of the model into the density map and is supported by the sharp turn of the backbone at the crossover junction. The C2′-*endo* conformation at this position of the crossover has previously been identified in similar crossovers in an RNA origami structure (3.4 Å local resolution) [42] and the hairpin ribozyme (2.2 Å resolution) [43]. The distortion of the helices results in bringing the 5′- and 3′-ends of the two different cmpRNAs into close proximity, with the 5′-end of one cmpRNA projecting above the 3′-end of the other cmpRNA (Fig. 2D).

### Twisting filaments produced by stacking of dimers

**Figure 3.**
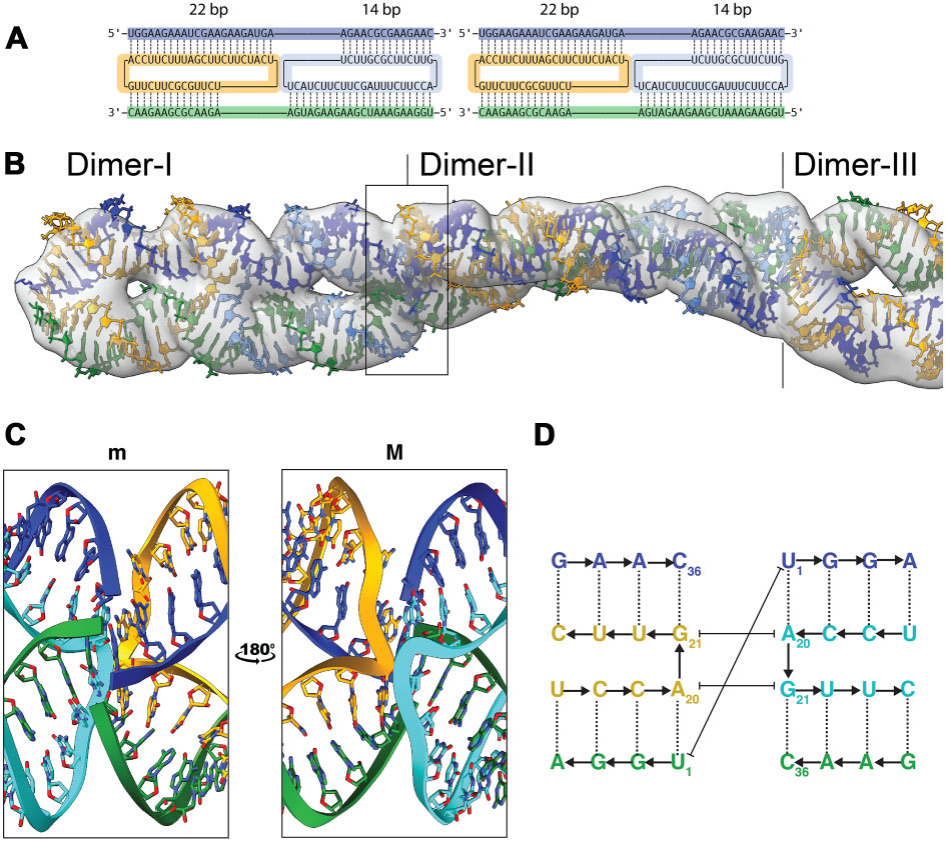
Multimeric structures composed of stacking homodimers. (**A**) Secondary structure of two homodimers attached end-to-end. (**B**) Cryo-EM map of class 5 with superimposed atomic models (EMDB: EMD-51934; PDB: 9H8A). (**C**) Zoom on stacking of the end-to-end crossover junction shown perpendicular to the C2 symmetry axis from the minor (m) and major (M) groove sides. (**D**) Secondary structure diagram showing the base stacking interactions between the terminal bases.

The density maps for classes 4 and 5 could be readily modeled by stacking class 3 dimers to form twisted filaments—with class 4 comprising two dimers and class 5 three dimers (Fig. 3A and B). These multimer models suggest that the interaction between dimer units in the filament is mediated by base stacking of terminal bases from each unit (Fig. 3C and D). Based on the models, we suggest that A20 and G21 of one circular strand stacked with G21 and A20 of the other circular strand, respectively, and the 5′-end U1 of one unit stack on the U1 of the second unit, while the 3′-ends were nonstacking. The 5′-ends point outward from the plane of the interaction and the 3′-ends point inward. The described distortion in the complex caused a global twist in the filament (period of ∼4 dimer units/turn) (Fig. 3B).

Notably, the 22–14 segmentation of the dimer also leads to a misalignment of the 5′- and 3′-ends of adjacent units. Indeed, this can be tested by ligation, since our structural model predicts that the dimers would ligate poorly. In contrast, scRNAs with a length corresponding to a whole integer of the RNA helical pitch (33 nt) would—despite also being distorted—likely present 5′- and 3′-ends better aligned for ligation. We tested this hypothesis using ligation experiments with dimers prepared from scRNAs and corresponding cmpRNAs that were either 33 or 36 nt in size (Supplementary Fig. S10 and Supplementary Table S1). We observed a more intense low-mobility ligation band for the 33-nt circle compared to the 36-nt circle, supporting our observed misalignment of the 5′- and 3′-ends in class 4 and 5 models.

### Partially hybridized structures

**Figure 4.**
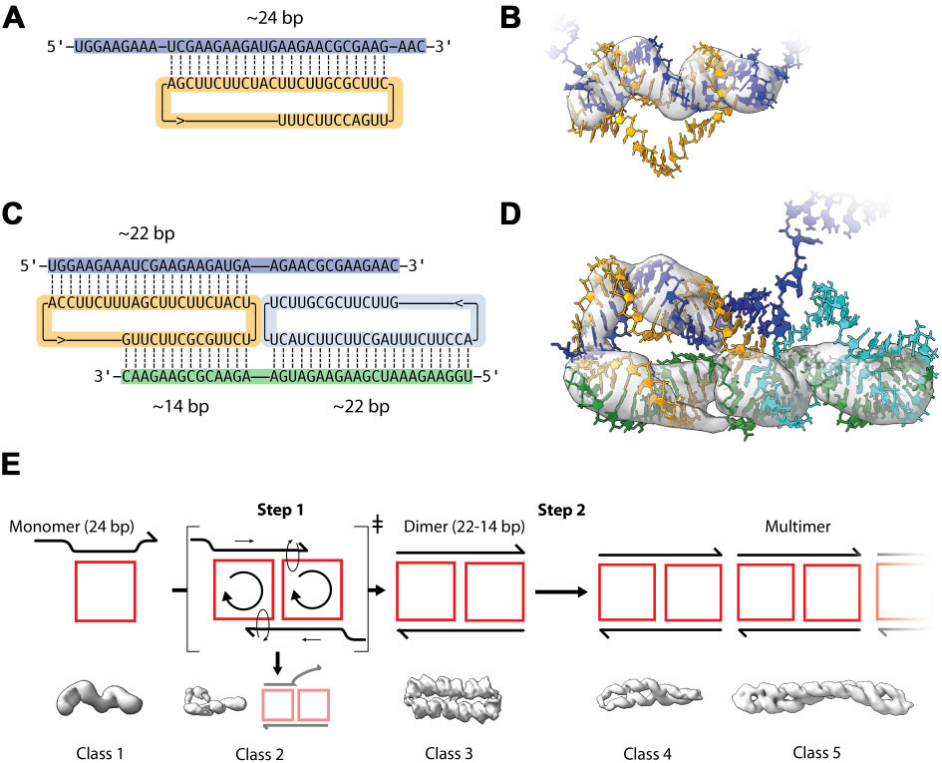
Partially hybridized complexes and hypothetical self-assembly path. (**A**) Secondary structure model of the scRNA bound to its product strand by ∼24 bp. (**B**) Density of cryo-EM class 1 superimposed on atomic model (EMDB: EMD-51918, PDB: 9H82). (**C**) Secondary structure model of two not fully hybridized scRNAs bound via their product strands. (**D**) Density of cryo-EM class 2 superimposed on atomic model (EMDB: EMD-51929; PDB: 9H83). (**E**) Schematics and density maps of the proposed assembly mechanism. Step 1 shows dimerization of class 1 monomers via a rolling circle mechanism leading to formation of the class 3 dimer. Step 2 shows multimerization of class 3 dimers by stacking to form class 4 and 5 multimers. Class 2 is proposed to represent a failed dimerization intermediate. The proposed assembly path is animated with morphing between atomistic models in Supplementary Movie S1.

The low-resolution cryo-EM maps of classes 1 and 2 are consistent with partly hybridized scRNA and cmpRNA. The density map for class 1 was observed to have slightly more than two helical turns. The density was analyzed by aligning a model of the scRNA–cmpRNA complex that was previously predicted by MD simulation to form ∼24 bp [29] (Supplementary Fig. S11). The similarity between the class 1 map and the MD model suggests that they could represent the same species. Based on this hypothesis, we constructed a new 3D model of the whole system that fits the class 1 density (Fig. 4A and B). Note that the cryo-EM density shows only the double-stranded segment, making the placement of the single-stranded regions ambiguous. The single-stranded regions are not seen in the map because they are flexible and are averaged out during 3D reconstruction. In Fig. 4A, we show the most G/C-rich 24-bp stretch since this will be the most thermodynamically stable structure; however, other base-pairing configurations are also compatible with the data.

The low-resolution density map for class 2 was consistent with a model composed of two scRNAs and two cmpRNAs, where the second cmpRNA had only partly hybridized. Based on the dimer model derived from class 3, we propose a model for class 2, where one cmpRNA is fully hybridized and the other cmpRNA is partly hybridized (Fig. 4C and D). Based on the position of the major grooves in the class 2 map, we can deduce that it is the 3′-end of one of the cmpRNAs that does not hybridize (as seen in Fig. 4C and D).

### Proposed self-assembly pathway for dimers and multimers

We propose that the structural classes 1–5 are all related and comprise a set of cmpRNA–scRNA complexes on a common folding path toward increasing thermodynamic stability. In class 1, only ∼66% of the possible base pairs are formed (∼24 bp out of 36 bp); in class 2, ∼80% are formed (∼58 bp out of 72 bp); and in class 3, 100% are formed (72 bp out of 72 bp). To explain the folding path for the formation of the observed species, we propose a rolling circle-based assembly mechanism (Fig. 4E). The folding path begins with the annealing of scRNA and cmpRNA to form class 1 monomers with a 24-bp annealing region. In step 1, two class 1 monomers dimerize and maximize base pairing by a rolling mechanism that weaves the strands together to form class 3. Rolling of the central crossover is known to occur in mobile Holiday junctions found in DNA recombination intermediates [44, 45]. Indeed, this conformation has been utilized to construct a DNA actuator [46, 47]. Several different base-pairing configurations of class 1 monomers can likely engage in the dimerization and rolling mechanism, but it can also be envisioned that some combinations of monomers get trapped. Interestingly, class 2 might represent a trapped state of the rolling mechanism, where only one of the unpaired ends has hybridized by the rolling mechanism (Fig. 4E, class 2). In step 2, class 3 dimers assemble into multimers by end-to-end stacking, leading to the filament-like class 4 and 5 structures. Our proposed assembly pathway is illustrated by morphing between atomistic models in Supplementary Movie S1.

### The dimer is both a roadblock and a substrate for RCS

**Figure 5.**
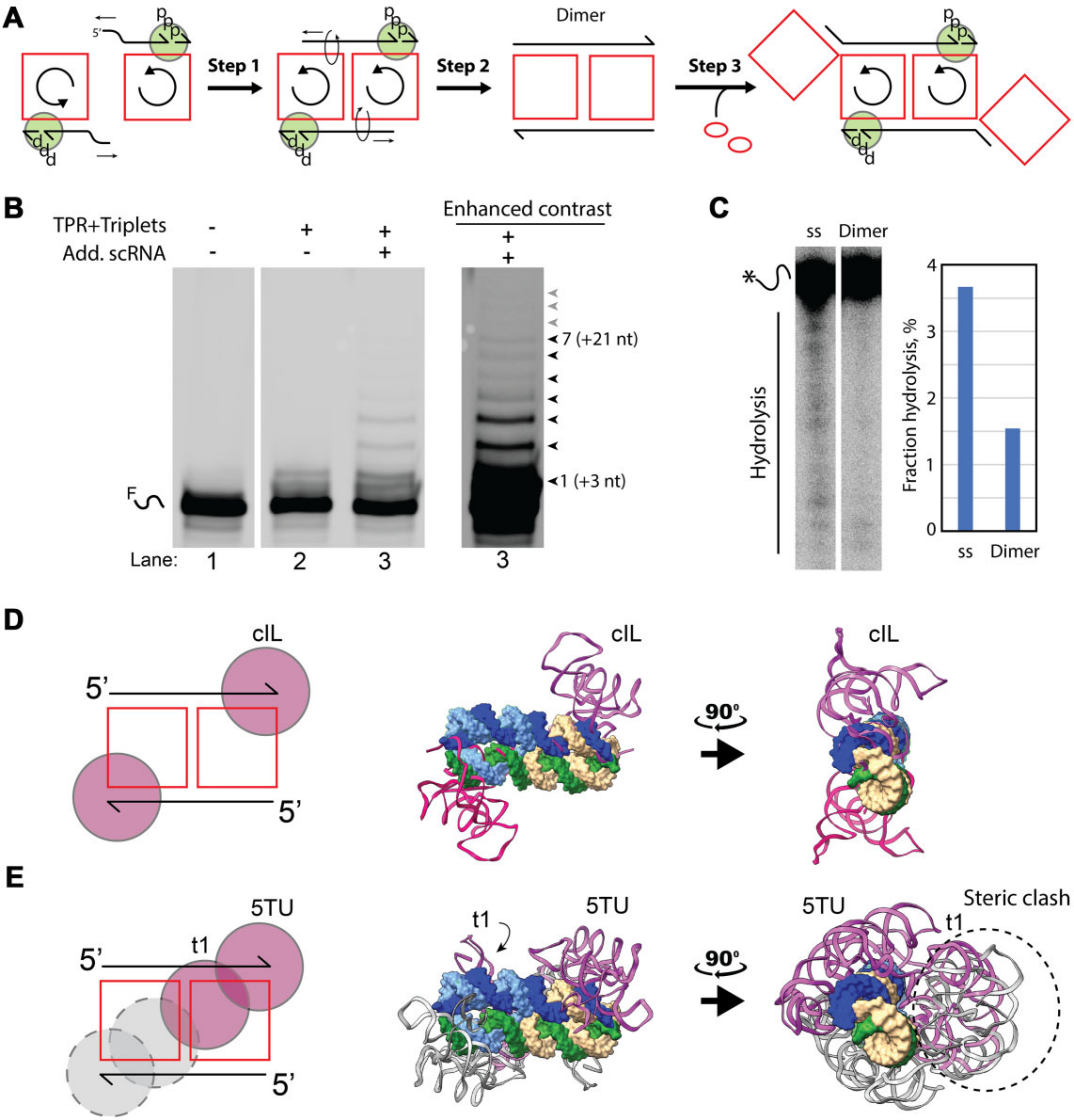
RCS and stability of dimer template. (**A**) Step 1 illustrates how two rolling circle replication complexes can dimerize by annealing of the displaced product strands to the single-stranded part of the scRNAs. Step 2 illustrates how continuous extension of the product strand can gradually extend the annealing region to form the inhibitory dimer. Step 3 illustrates how the addition of more scRNAs can allow continued synthesis (see Supplementary Movie S2). The circle symbolizes the TPR and ppp represents the triphosphates on the triplet nucleotides. (**B**) Primer extension analysis by TPR on denaturing gel. (**C**) Denaturing gel (10%, 19:1) shows hot-labeled cmpRNA hydrolysis after incubation (1 week at −7°C) under reaction conditions of single-stranded RNA (ss) and dimer. Bar chart shows quantified hydrolysis measured as band intensity under the top full-length band. (**D**) Model docking showing how two class I ligase (cIL) ribozymes can dock onto the dimer with the active sites positioned to ligate a triplet substrate. On the left is a cartoon illustration of the dimer (squares and lines) as well as the cIL (circles), and on the right is the full atomistic model with the dimer (shown as surface) and the cIL (shown as cartoon). (**E**) Same as in panel (D) but with TPR (double circle) instead of cIL. Due to steric clash the second TPR is theoreticallyunable to bind (dashed double circle).

Our structural study of scRNA and cmpRNA hybridization revealed an unexpected diversity of structures that represent likely RNA-catalyzed RCS intermediates as the nascent strand is extended toward full length (Fig. 5A, steps 1 and 2). The structure of the class 3 dimer suggests that it may act as a dead-end product for RCS by inhibiting 3′-end extension due to its thermodynamic stability and poor accessibility of 3′-ends, providing a plausible candidate for the previously observed full-length synthesis inhibition [29]. To test this, we gel-purified class 3 dimer and investigated whether the cmpRNA 3′-end could be extended by TPR. As predicted, primer extension analysis on denaturing gel showed that the cmpRNA could not be extended, when it was part of the dimer complex (Fig. 5B, lane 2), even upon prolonged incubation (Supplementary Fig. S12). This result strongly suggests that both the strong RCS inhibition observed at one round of scRNA extension [29] and the observation that dilution decreased this inhibitory effect [29] are due to the formation of a class 3 dimer, since dilution may slow dimer formation resulting in more class 1 monomer structures compatible with RCS.

The rolling circle assembly mechanism shown in Fig. 4E led us to the hypothesis that a similar (but reverse) rolling mechanism could allow the class 3 dimer to be a substrate for RCS by the annealing and iterative polymerization of new triplets by TPR on both scRNA templates (see the proposed mechanism in Supplementary Movie S2). Inspired by the controlled rolling motion of a DNA actuator by the addition of extra DNA strands [46, 47], we hypothesized that the replication block might be relieved by addition of extra scRNA templates to allow both cmpRNAs of the dimer to slide and facilitate additional templated elongation of their 3′-ends (Fig. 5A, step 3). Indeed, the addition of extra scRNA templates (2:1, circle relative to dimer) restarted the extension reaction of the previously inert dimer even though with modest efficiency (Fig. 5B, lane 3). We propose that this continued extension of the 3′-end is facilitated by the hybridization of the 5′-ends to the scRNAs and the triplet substrates to the scRNA templates at the 3′-end of the cmpRNAs (Fig. 5A, step 3). A similar mechanism might be driven by internal folding of the nascent 5′ region (i.e. cotranscriptional folding).

### Roles of dimer in stability and multimerization

The fully double-stranded nature of the dimer structure suggested that it would likely have an increased chemical stability. To investigate this, we formed dimer complexes with hot-labeled cmpRNA and incubated it for 1 week under reaction conditions at −7°C to allow hydrolysis to occur; the cmpRNA was incubated either as a single-stranded RNA or as part of the dimer. Analysis on denaturing gel showed that the cmpRNA was protected from hydrolysis, being over three-fold more stable, when it was part of the dimeric complex as compared to being single-stranded (Fig. 5C). This observation is in agreement with the fact that single-stranded RNA has a tendency to self-hydrolyze due to the nucleophilic activity of the 2′-OH group in the presence of magnesium. When the RNA is folded in a rigid RNA structure, the 2′-OH groups are fixed and prevented from reacting with the phosphodiester bond. Thus, a structured template has the benefit of being more stable and long-lived, and we propose that the dimer could serve as a stable ‘‘storage’’ form for RNA replication products in a prebiotic scenario.

The stable structure of the dimer furthermore allows multimers to form by end-to-end stacking. As described in Fig. 3, the interaction is not sequence-specific but rather due to the precise strained geometry of the dimer caused by the length of the circle. We therefore predict that the 36-nt circle dimers will preferentially multimerize with copies of themselves. This would lead to filaments of the same circular species and might provide a simple form of genetic segregation to keep the same kind of circular genomes bound together.

### Structural modeling of RCS in the dimer complex

To investigate the proposed ‘‘double RCS’’ further, we docked the structure of the cIL ribozyme (PDB: 3IVK) and TPR (PDB: 8T2P) onto the structural model of the class 3 dimer (PBD: 8S6W), so the active sites of the ribozymes were positioned on the dimer at the 5′-end of an annealed triplet. The model showed that cIL could access the ligation junction (Fig. 5D), while TPR could only be bound one at a time, since the binding of two TPRs at the two ends of the dimer simultaneously was hindered due to a steric clash of the noncatalytic t1 subunits (Fig. 5E). The steric clash is likely part of the reason for the low efficiency of the extension on the dimer by TPR, but this might be solved in the future through new and improved RNA polymerase ribozymes selected for circRNA templates. Indeed, cooperative 3′-end extension by two ribozymes might provide sufficient energetic driving force for a double rolling scRNA mechanism without the addition of additional scRNA templates for activation.

As suggested by the structure of the dimer, the 5′-end of one cmpRNA projects above the 3′-end of the other cmpRNA (Fig. 2D), thus priming the complex to roll in the correct 3′–5′ direction and inhibit rolling in the reverse (5′–3′) direction. Reverse rolling is also prevented by the polymerization of triplets at the 3′-end. An interesting property of the dimer arises if the circular template encodes an RNA polymerase ribozyme. In this case, the nascent strand may fold into an active RNA polymerase ribozyme that has the potential to act on itself (in *cis*) within the replication complex (Supplementary Fig. S13B). For the monomer RCS, the distance between the 5′-end and the position of the ligation is 8 nm for a 36-nt template and increases with template length. However, for the dimer RCS, the distance between the 5′-end and the nearest ligation site is only 4 nm and does not increase with template length.

### Potential benefits of dimer in proofreading and template size

The rolling mechanism of the dimer might enhance RCS fidelity by disfavoring base misincorporation (including bases with noncognate chemistry or chirality) (Supplementary Fig. S13C) through stalling and enhanced hydrolysis. As the rolling relies on the isoenergetic relation between 3′-end base pair formation and base pair melting in the 5′-end, incorporation of mismatched bases at the 3′-end will destabilize 3′-end hybridization and in turn disfavor coordinated 5′-end melting and thus inhibit extension as well as potentially expose mismatched, single-stranded 3′-ends to increased hydrolysis. Such a mechanism could make sure that only ‘‘proof-read’’ sequences would make it through the “cogwheels” of the structure and get extruded in the 5′-end. Finally, because this RCS would be a coordinated process, misincorporation on one scRNA would also inhibit further extension on the second scRNA, increasing fidelity of replication on both templates simultaneously. These potential features of RCS merit further investigation.

We have investigated the properties of a 36-nt circular template, but many different sizes of RNA circles could conceivably be investigated for their properties in forming the dimer and their function as a template for two-fold RCS. We would predict that scRNAs in the length range of 22 to several hundred bp would likely form dimer-type structures (at least to some extent) due to the persistence length of the double-stranded RNA helix of ∼300 bp [30]. We also show that these dimers must be either avoided or reactivated to support continuous RCS. Taking these factors into consideration in further experiments as well as in engineering future polymerase ribozymes might lead to more efficient RCS.

## Conclusion

In this study, we have used cryo-EM to investigate the RCS process on scRNA templates by the structural analysis of putative RCS intermediates and products. We find that scRNAs and cmpRNAs of 36 nt in length can form an unanticipated diversity of structures with different stoichiometries, including a fully annealed dimer (comprising two scRNAs and two cmpRNAs) and multimers hereof. These complexes form because of the restrictions on hybridization of scRNA templates and the cmpRNAs, which arise due to geometric strain in the scRNA when the circle size is considerably smaller than the persistence length of duplex RNA. Furthermore, we show that the dimer structure is a highly stable dead-end complex that inhibits the primer extension by the TPR, but that inhibition can be relieved by addition of additional scRNAs. We propose a new double-circle RCS mechanism based on the dimer structure and its ability to be extended by TPR in the presence of additional scRNA templates. Our study further underlines the importance of the template structure and topology in RNA-catalyzed replication and outlines how rolling circle replication mechanisms might have provided fitness benefits in prebiotic RNA replication.

## Supplementary Material

gkaf057_Supplemental_Files

## Data Availability

Cryo-EM maps were deposited in the Electron Microscopy Data Bank under accession numbers EMD-51918 (class 1), EMD-51929 (class 2), EMD-19759 (class 3), EMD-51932 (class 4), and EMD-51934 (Class 5). Atomic coordinates have been deposited into the PDB under accession numbers 9H82 (class 1), 9H83 (class 2), 8S6W (class 3), 9H86 (class 4), and 9H8A (class 5). The raw data were deposited to EMPIAR [48] under accession number EMPIAR-12173 (https://doi.org/10.6019/EMPIAR-12173).
