# Supplementary material for: Roles of dimeric intermediates in RNA-catalyzed rolling circle synthesis
Source: Nucleic Acids Res. 2025 Jun 16;53(11):gkaf057. doi: 10.1093/nar/gkaf057 (PMC12168072; doi:10.1093/nar/gkaf057)
Supplement: gkaf057_Supplemental_Files [file gkaf057_supplemental_files.zip › Dimer-circle-SM_new.docx]

**Supplementary material**

**Roles of dimeric intermediates in RNA-catalyzed rolling circle synthesis**

Emil L. Kristoffersen^1, 2, 3^, Ewan K. McRae^1, 3^, Niels R. Sørensen^1^, Philipp Holliger^2, 4^*, Ebbe S. Andersen^1, 4^*

^1^Interdisciplinary Nanoscience Center, Aarhus University, Aarhus, Denmark; ^2^MRC Laboratory of Molecular Biology, Francis Crick Avenue, Cambridge CB2 0QH, UK

*Communication to: [ph1@mrc-lmb.cam.ac.uk](mailto:ph1@mrc-lmb.cam.ac.uk) & [esa@inano.au.dk](mailto:esa@inano.au.dk)

**Content:**

[Table S1. Nucleic acid sequences 2](#_Toc183426893)

[Table S2. Cryo-EM data collection, refinement and validation statistics 4](#_Toc183426894)

[Figure S1. Raw gel images. Boxes indicate regions used in main text figures. 5](#_Toc183426895)

[Figure S2*.* Representative Cryo-EM 2D class averages from cryoSPARC Live. 6](#_Toc183426896)

[Figure S3. Cryo-EM workflow for monomer species (class 1-3). 7](#_Toc183426897)

[Figure S4. Cryo-EM workflow for intermediates (class 4-5). 8](#_Toc183426898)

[Figure S5. Density map parameters - class 1 (EMD-51918). 9](#_Toc183426899)

[Figure S6. Density map parameters - class 2 (EMD-51929). 10](#_Toc183426900)

[Figure S7. Density map parameters - class 3 (EMD-19759). 11](#_Toc183426901)

[Figure S8. Density map parameters - class 4 (EMD-51932). 12](#_Toc183426902)

[Figure S9. Density map parameters - class 5 (EMD-51934). 13](#_Toc183426903)

[Figure S10. Gel analysis of scRNA length, assembly, and ligation. 14](#_Toc183426904)

[Figure S11. Fitting of MD model to class 1 density. 15](#_Toc183426905)

[Figure S12. Primer extension scRNA species by TPR. 16](#_Toc183426906)

[Figure S13. Dimeric rolling circle replication hypothesis. 17](#_Toc183426907)

# Table S1. Nucleic acid sequences

| ***Oligo name*** | ***Seq. (5' to 3')*** | ***type*** | ***Notes:*** |
| --- | --- | --- | --- |
| **Templates:** |  |  |  |
| **scRNA** | /5Phos/GCG UUC UUC AUC UUC UUC GAU UUC UUC CAG UUC UUC | RNA | Used as either linear or circularized. |
| **cmpRNA** | UGG AAG AAA UCG AAG AAG AUG AAG AAC GCG AAG AA /3ddC/ | RNA | When noted this sequence was 5’ hot labelled. |
| **F-cmpRNA** | /56-FAM/GAA GAA CUG GAA GAA AUC GAA GAA GAU GAA GAA CGC | RNA |  |
| **P9** | /56-FAM/GAA GAA CUG | RNA |  |
| **33 nt circle  (11-GAC)** | GATCGATCTCGCCCGCGAAATTAATACGACTCACTATA-*GTCGTCGTCGTCGTCGTCGTCGTCGTCGTCGTC*-GGGTCGGCATGGCATC | DNA | Fill-in with HDVrt (as back (Ba) primer) and in vitro transcribe. This leads to production of the *italic sequence* as the reverse compliment RNA product with the HDV ribozyme cleaved off. Cyclic phosphate was removed with PNK. |
| **36 nt circle  (12-GAC)** | GATCGATCTCGCCCGCGAAATTAATACGACTCACTATA-*GTCGTCGTCGTCGTCGTCGTCGTCGTCGTCGTCGTC*-GGGTCGGCATGGCATC | DNA | Fill-in with HDVrt (as back (Ba) primer) and in vitro transcribe. This leads to production of the *italic sequence* as the reverse compliment RNA product with the HDV ribozyme cleaved off. Cyclic phosphate was removed with PNK. |

| **Oligonucleotides for synthesis of triplet RNA Polymerase Ribozyme (TPR):** |  |  |  |
| --- | --- | --- | --- |
| **5TU (Fo fill-in)** | GGATCTTCTCGATCTAACAAAAAAGACAAATCTGCCACAAAGCTTGAGAGCATCTTCGGATGCAGAGGCGGCAGCCTTCGGTGGCGCGATAGCGCCAACGTTCTCAACTATGACACGCAA | DNA |  |
| **5TU (Ba fill-in)** | CTTCTCCCTTAGCCTACCGAAGTAGCCCAGGTCGGACCGCGAGGAGGTGGAGATGCCATGCCGACCCCATGATAAACTCCATTCAACGGAGCACGCGTTTTGCGTGTCATAGTTGAGAAC | DNA |  |
| **t1 (Fo fill-in)** | GACCAATCTGCCCTCAGAGCTCGAGAACATCTTCGGATGCAGAGGAGGCAGGCTTCGGTGGCGCGATAGCGCCAACGTCCTCAACCTCCAATGCATCCCACCACATGATGATGCCTGAAG | DNA |  |
| **t1 (Ba fill-in)** | CTTCTCCCTTAGCCTACCGAAGTAGCCCAGGTCGGACCGCGAGGAGGTGGAGATGCCATGCCGACCCCAAAAAACCAAGGCTCTTCAGGCATCATCATGTG | DNA |  |
| **5TU (final RNA product)** | GGAUCUUCUCGAUCUAACAAAAAAGACAAAUCUGCCACAAAGCUUGAGAGCAUCUUCGGAUGCAGAGGCGGCAGCCUUCGGUGGCGCGAUAGCGCCAACGUUCUCAACUAUGACACGCAAAACGCGUGCUCCGUUGAAUGGAGUUUAUCAUG | RNA | To make: Fill-in with 5TU Fo and Ba fill-in primers, then PCR with t5T7pFo and HDVrt. |
| **t1 (final RNA product)** | GACCAAUCUGCCCUCAGAGCUCGAGAACAUCUUCGGAUGCAGAGGAGGCAGGCUUCGGUGGCGCGAUAGCGCCAACGUCCUCAACCUCCAAUGCAUCCCACCACAUGAUGAUGCCUGAAGAGCCUUGGUUUUUUG | RNA | To make: Fill-in with t1 Fo and Ba fill-in primers, then PCR with t1T7pFo and HDVrt. |

| **Primers:** |  |  |  |
| --- | --- | --- | --- |
| **5T7 (Fo)** | GATCGATCTCGCCCGCGAAATTAATACGACTCACTATA | DNA |  |
| **HDVrt (Ba)** | CTTCTCCCTTAGCCTACCGAAGTAGCCCAGGTCGGACCGCGAGGAGGTGGAGATGCCATGCCGACCC | DNA |  |

# Table S2. Cryo-EM data collection, refinement and validation statistics

|  | #1 Class 1  (EMD-51918)  (PDB 9H82) | #2 Class 2  (EMD-51929)  (PDB 9H83) | #3 Class 3  (EMD-19759)  (PDB 8S6W) | #4 Class 4  (EMD-51932)  (PDB 9H86) | #5 Class 5  (EMD-51934)  (PDB 9H8A) |
| --- | --- | --- | --- | --- | --- |
| **Data collection and processing** |  |  |  |  |  |
| Magnification | 130,000 | 130,000 | 130,000 | 130,000 | 130,000 |
| Voltage (kV) | 300 | 300 | 300 | 300 | 300 |
| Electron exposure (e–/Å^2^) | 60 | 60 | 60 | 60 | 60 |
| Defocus range (μm) | -2.0 to 0.8 | -2.0 to 0.8 | -2.0 to 0.8 | -2.0 to 0.8 | -2.0 to 0.8 |
| Pixel size (Å) | 2.7 | 2.7 | 2.7 | 2.7 | 2.7 |
| Symmetry imposed | No | No | C2 | No | No |
| Initial particle images (no.) | 668,921 | 668,921 | 668,921 | 668,921 | 668,921 |
| Final particle images (no.) | 48117 | 48761 | 117663 | 24380 | 15694 |
| Map resolution (Å)  FSC threshold | 7.29 0.143 | 7.8 0.143 | 5.1 0.143 | 9.3 0.143 | 9.6 0.143 |
| Map resolution range (Å)   (min-75th percentile) | 6.5-9.5 | 7.0-8.5 | 4.7-7.4 | 8.6-9.9 | 9.9-11.1 |
|  |  |  |  |  |  |
| **Refinement** |  |  |  |  |  |
| Initial model used (PDB code) | NA | NA | NA | NA | NA |
| Model resolution (Å)  FSC threshold | 10.3  0.143 | 10.5 0.143 | 5.1 0.143 | 10.3  0.143 | 10.7  0.143 |
| Map sharpening *B* factor (Å^2^) | None | None | -293 | None | None |
| Model composition  Non-hydrogen atoms  Nucleotide residues  Ligands | 1238  58  - | 2305  109  - | 3054  144  - | 6108  288  - | 6108  288  - |
| R.m.s. deviations  Bond lengths (Å)  Bond angles (°) | 0.014 (1)  2.369 (37) | 0.013 (0)  1.821 (47) | 0.014 (0)  1.721 (22) | 0.013 (0)  1.743 (75) | 0.013 (0)  1.743 (75) |
| Validation  MolProbity score  Clashscore | 1.96  1.07 | 1.98  1.15 | 1.65  0.00 | 1.69  0.11 | 1.69  0.11 |
| Cross-correlation score | 0.82 | 0.81 | 0.91 | 0.84 | 0.91 |


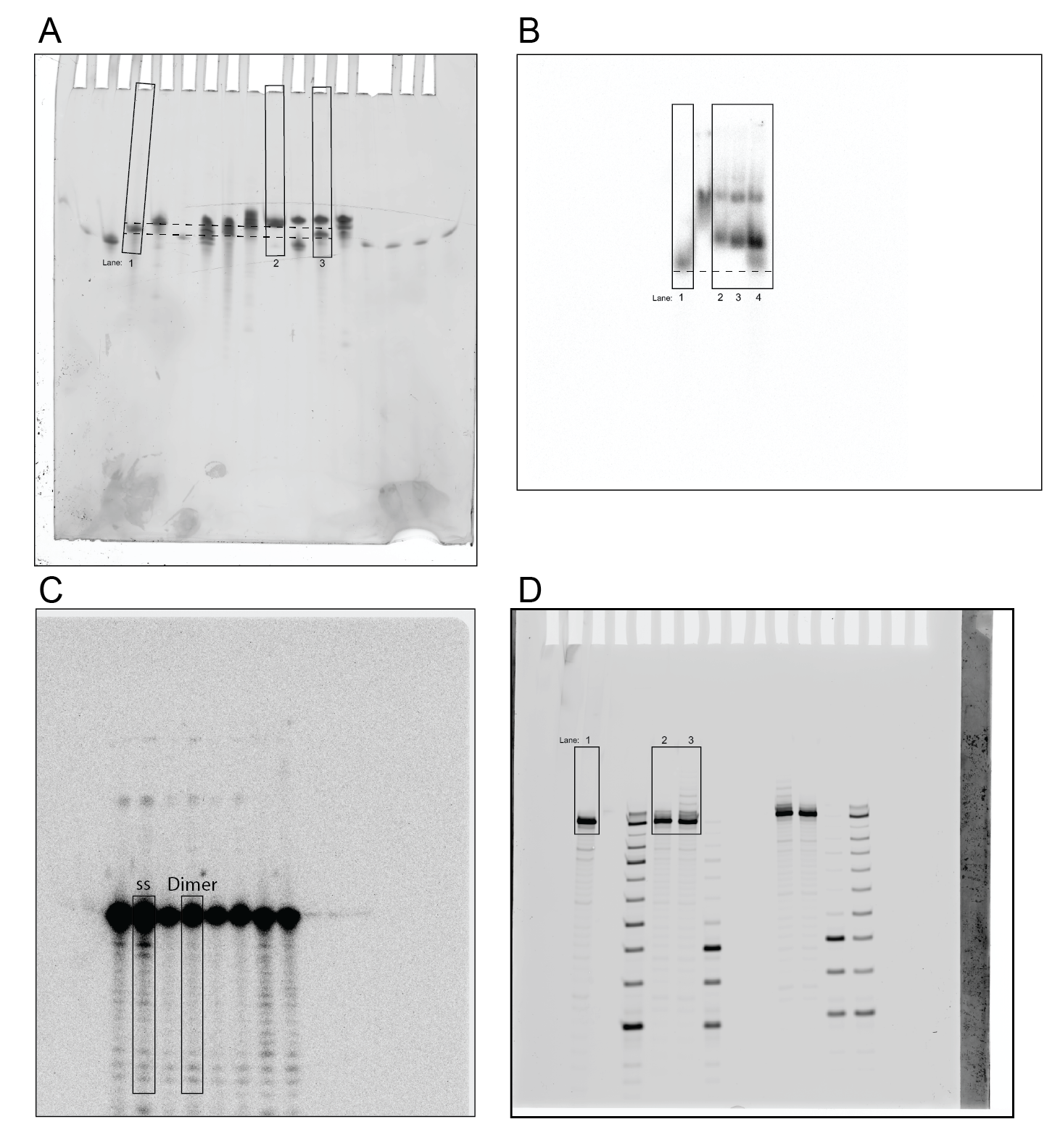


# Figure S1. Raw gel images. Boxes indicate regions used in main text figures.

(A) 8 M urea denaturing gel (20%, 19:1) from Figure 1B. (B) Native gel (10%, 37:1) from Figure 1C. (C) Denaturing gel (10%, 19:1) from Figure 5C. (D) Denaturing gel (20%, 19:1) from Figure 5B. Boxed lanes are used in the main figures.


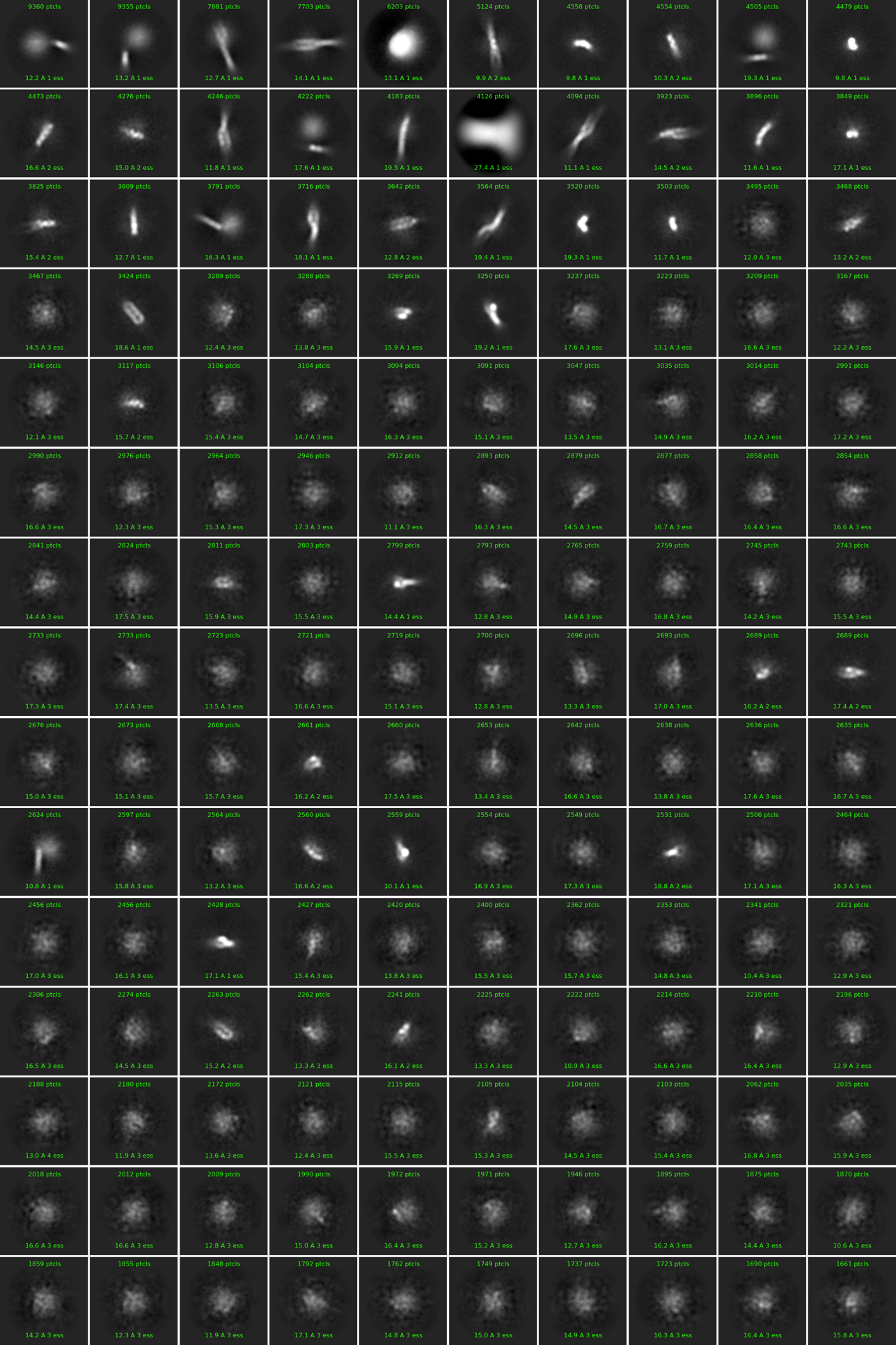


# Figure S2*.* Representative cryo-EM 2D class averages from cryoSPARC Live.


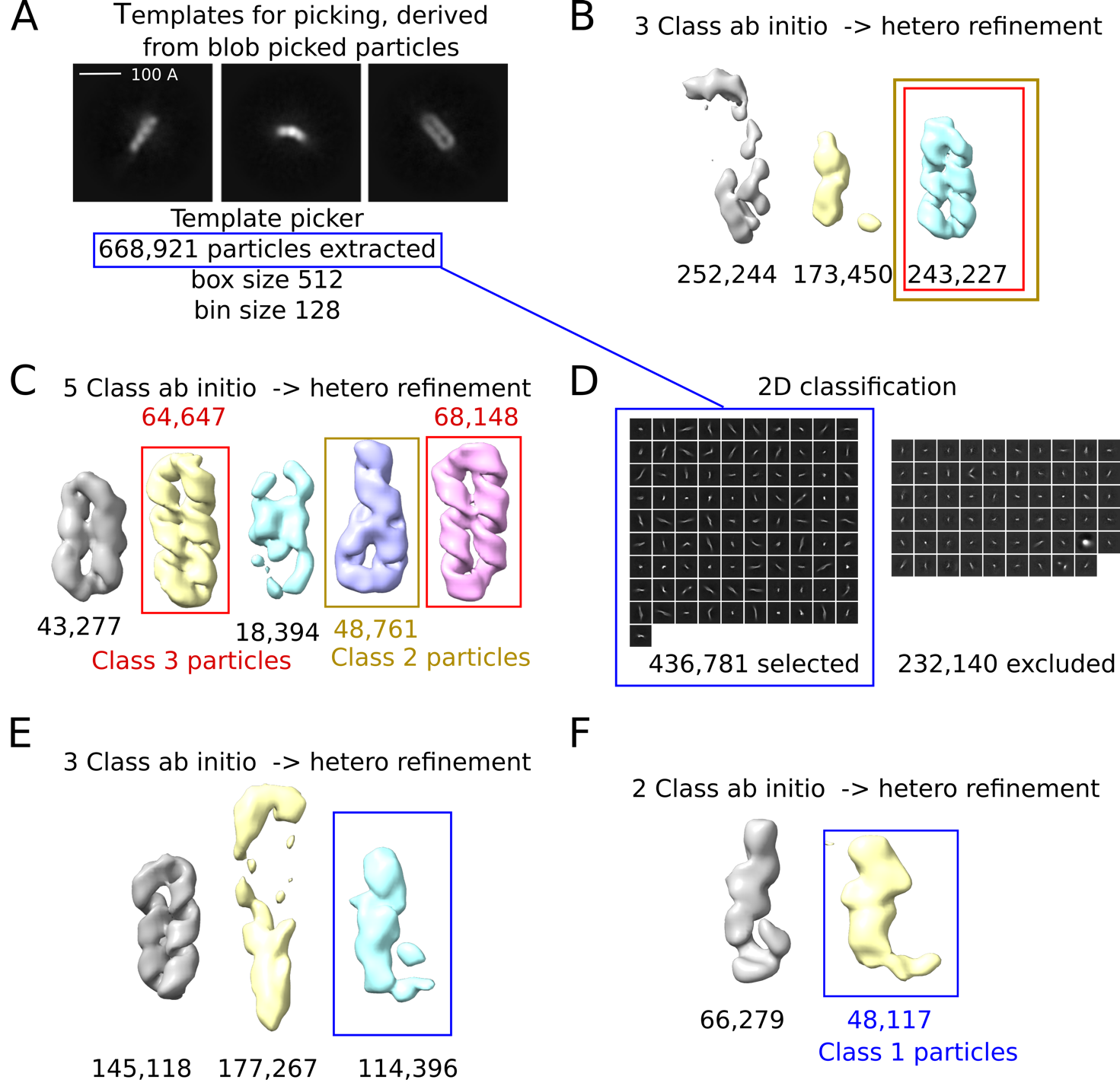


# Figure S3. Cryo-EM workflow for monomer and dimer species (class 1-3).

Following cryoSPARC Live processing, 2D class averages were used as templates for particle picking (A). Ab initio reconstruction and heterogeneous refinement identified class 3-like particles (B). These class 3-like particles were further split into 5 classes (C) resulting in ~49k particles for class 2 (burnt golden rod) and ~117k particles for class 3 (red). 2D classification was used to select for higher signal particles (D). This allowed for a two-step ab initio sorting approach, first with 3 classes (E) and then with 2 classes (F) to identify particles which reconstruct to give class 1 (blue).


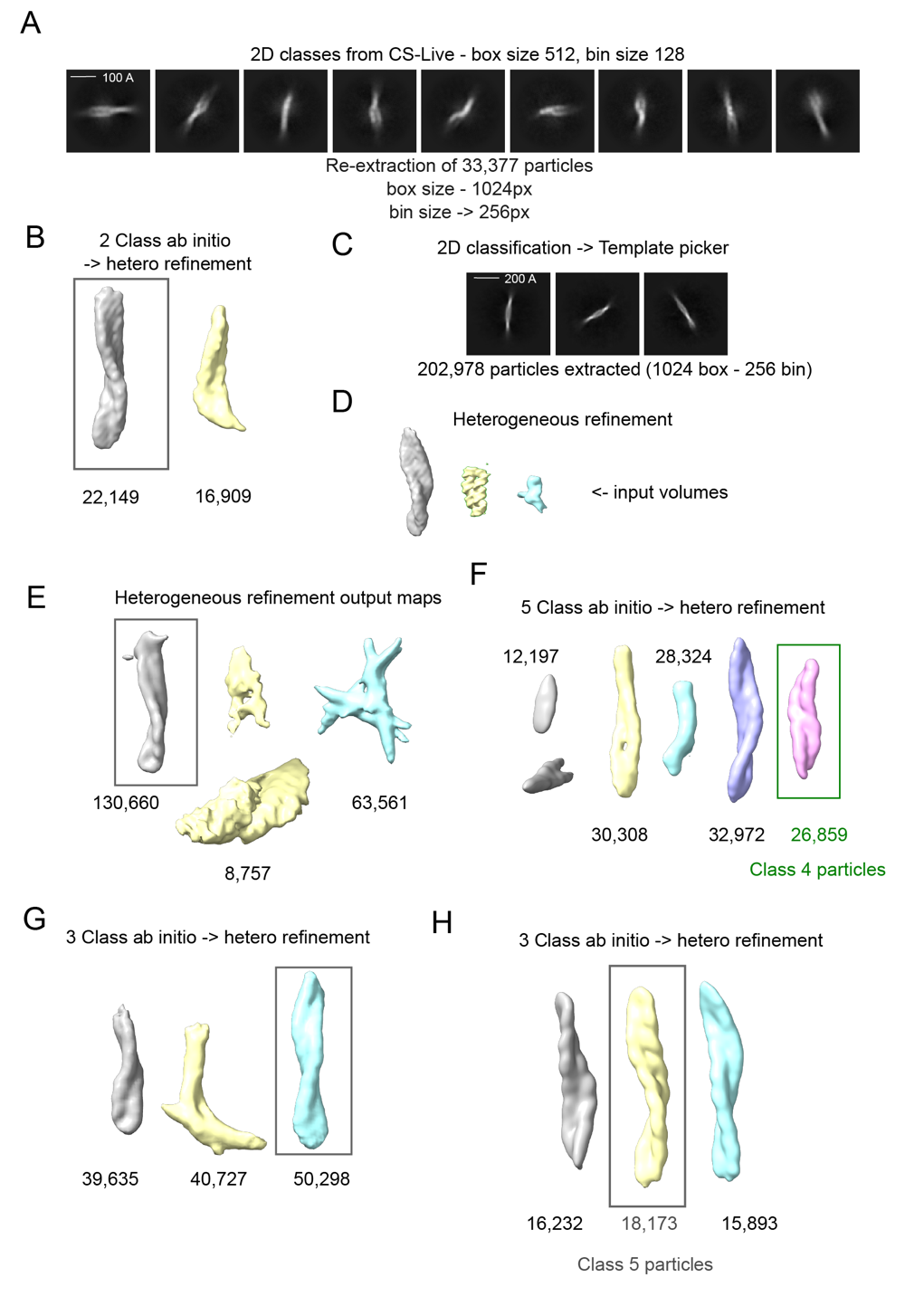


# Figure S4. Cryo-EM workflow for multimeric species (class 4-5).

Extended particles from cryoSPARC Live generated 2D class averages were re-extracted with a bigger box (A) and used for ab initio reconstruction (B). 2D class averages from the boxed particles in B (C) were used for templated particle picking. Three input volumes (D) were used to sort the resulting particles in 3D (E). The 130,000 particles sorted into the extended class were used for 5-class (F) and 3-class (G) ab initio reconstruction followed by heterogeneous refinement. The 5-class sorting (F) identified particles that reconstruct to give a length equivalent to two of class 3. The 3-class sorting (G) identified a longer filament approximately equivalent to 2.5-3 of class 3. This longer class was further sorted by an additional round of 3-class ab initio reconstruction (H) followed by heterogeneous refinement.


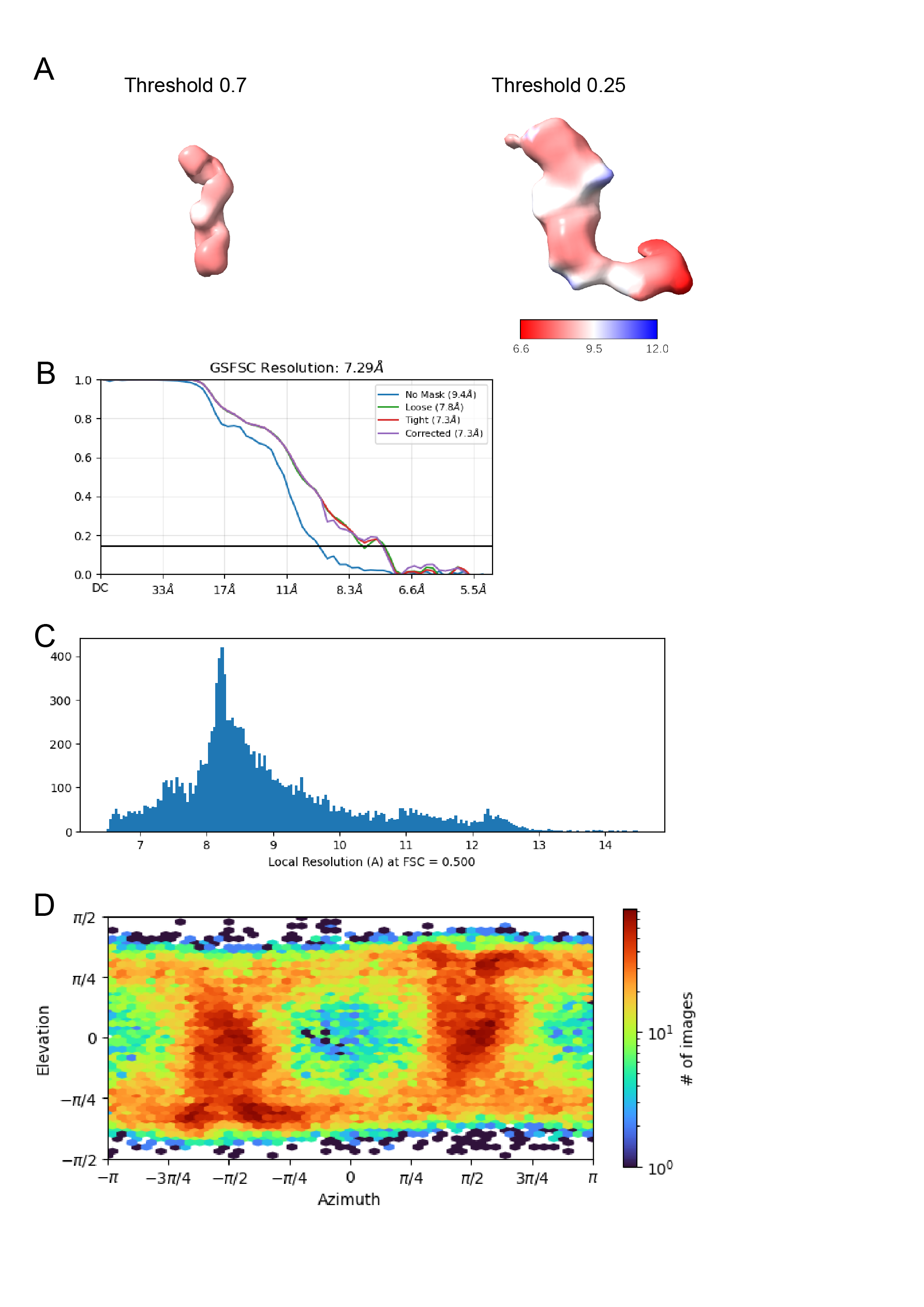


# Figure S5. Density map parameters - class 1 (EMD-51918).

Local resolution estimation using default parameters in cryoSPARC and visualized in ChimeraX at 2 different thresholds (A). Final Gold-Standard Fourier Shell Correlation plot (B). Local resolution histogram (C). Angular distribution of final particle alignments (D).


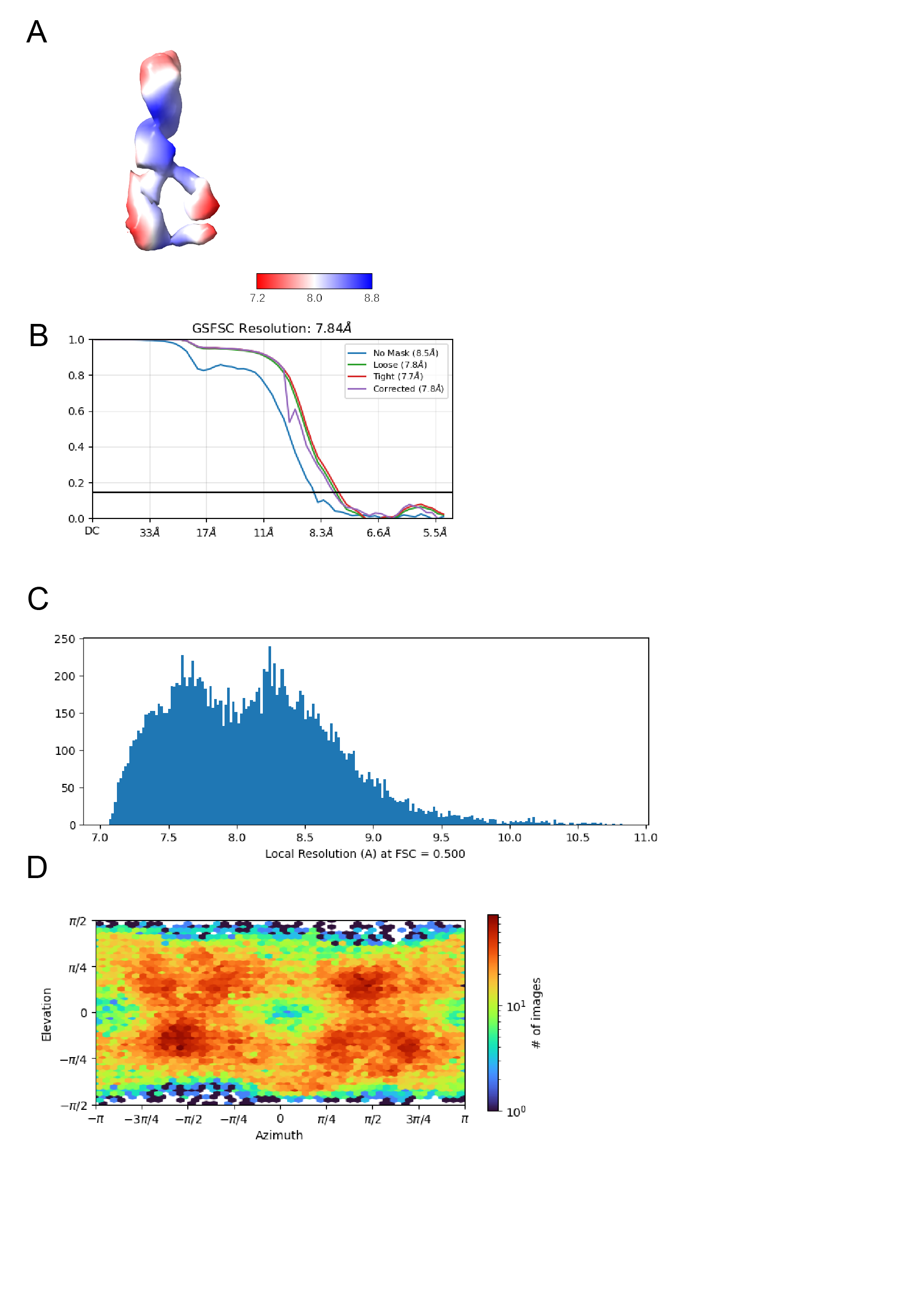


# Figure S6. Density map parameters - class 2 (EMD-51929).

Local resolution estimation using default parameters in cryoSPARC and visualized in ChimeraX (A). Final Gold-Standard Fourier Shell Correlation plot (B). Local resolution histogram (C). Angular distribution of final particle alignments (D).


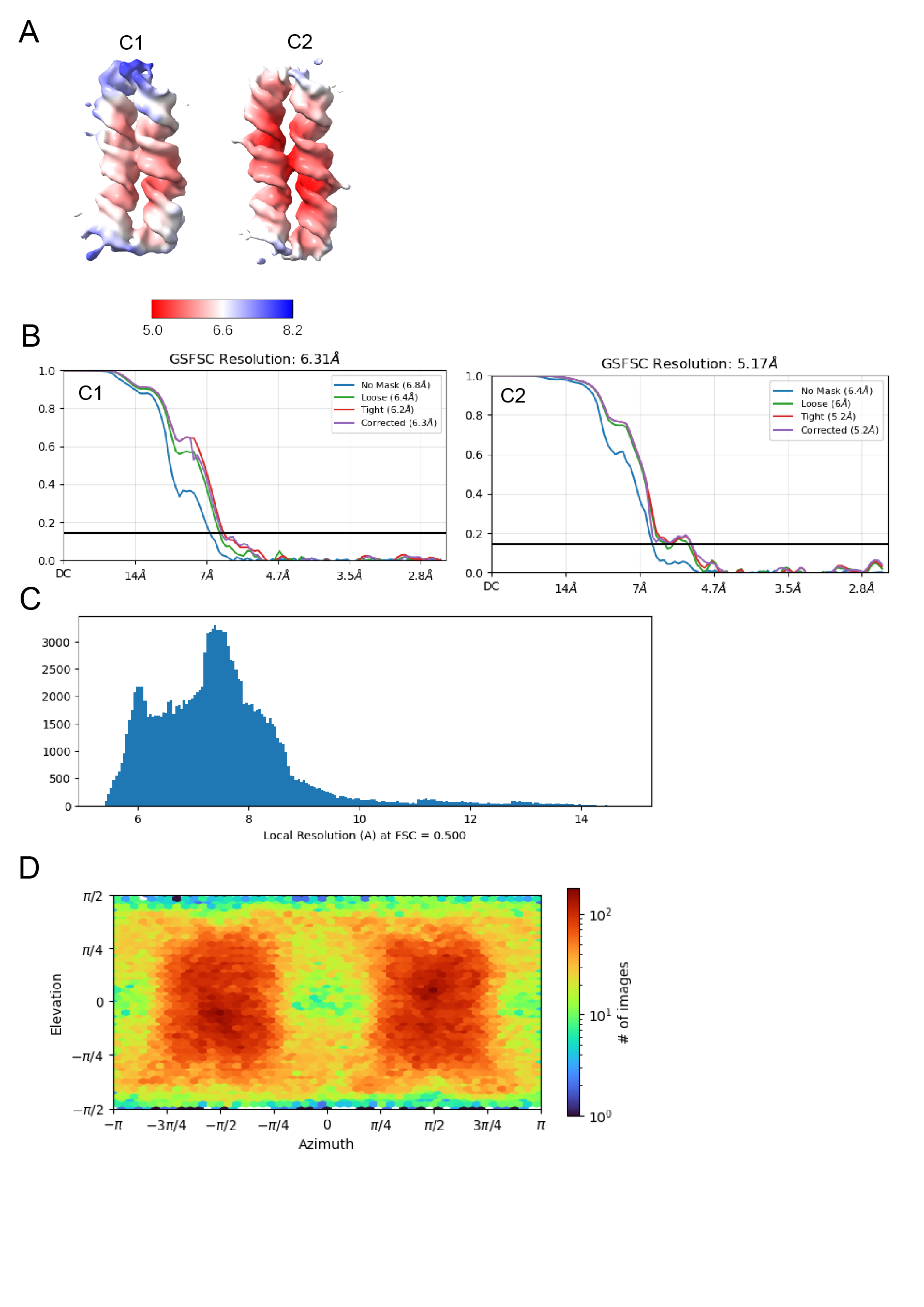


# Figure S7. Density map parameters - class 3 (EMD-19759).

Local resolution estimation using default parameters in cryoSPARC and visualized in ChimeraX (A). Final Gold-Standard Fourier Shell Correlation plots (B). Local resolution histogram for C1 (C). Angular distribution of final particle alignments of C1 (D)**.**


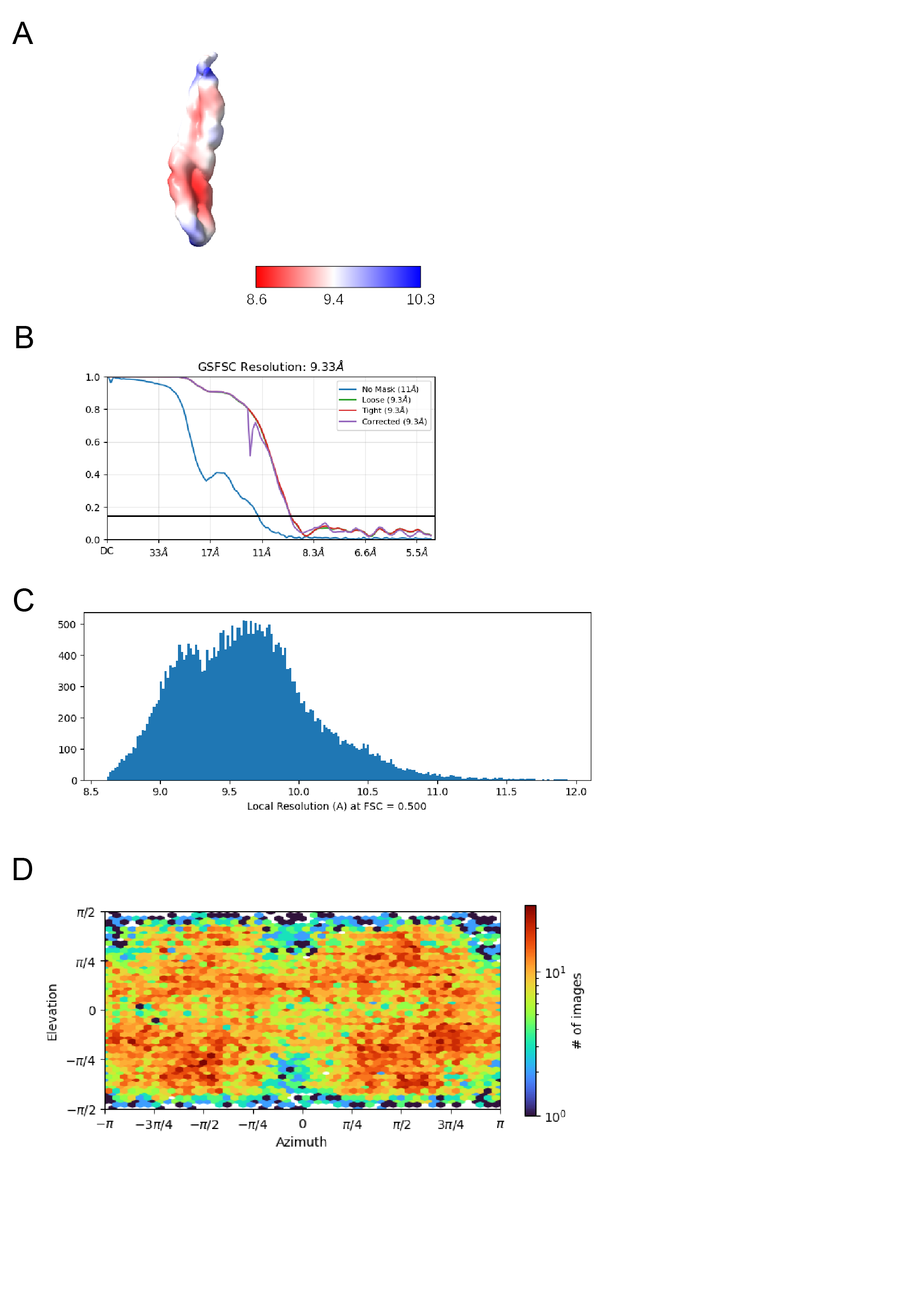


# Figure S8. Density map parameters - class 4 (EMD-51932).

Local resolution estimation using default parameters in cryoSPARC and visualized in ChimeraX (A). Final Gold-Standard Fourier Shell Correlation plot (B). Local resolution histogram (C). Angular distribution of final particle alignments (D)**.**


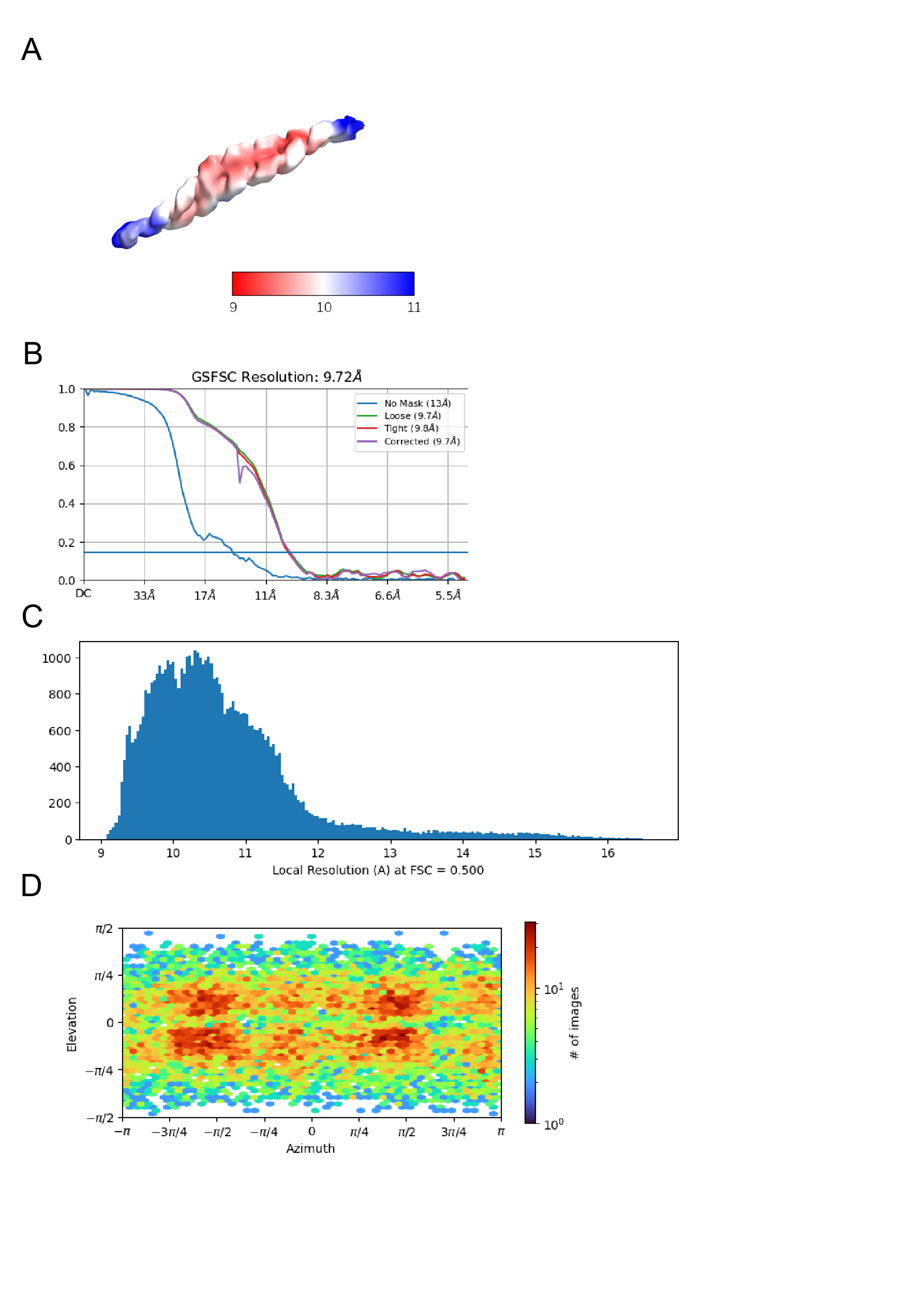


# Figure S9. Density map parameters - class 5 (EMD-51934).

Local resolution estimation using default parameters in cryoSPARC and visualized in ChimeraX (A). Final Gold-Standard Fourier Shell Correlation plot (B). Local resolution histogram (C). Angular distribution of final particle alignments (D)**.**

**
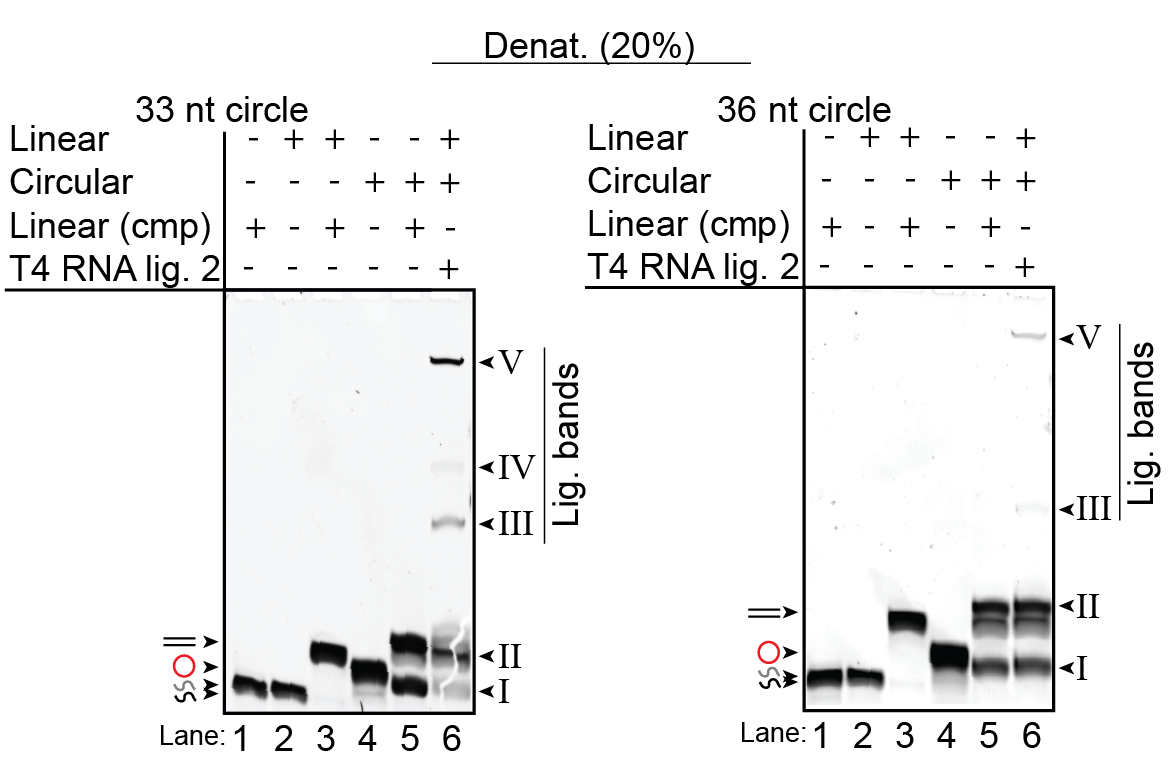
**

# Figure S10. Gel analysis of scRNA length, assembly, and ligation.

Denaturing (20%, 19:1) gels of RNA strands with varying sequence and length (SYBR Gold stained). Only the stable conformations are stable under the denaturing conditions. Upon incubation of linear cmpRNA with the circular template (lane 5) stable complexes were observed (band II) similar to gels shown in main text Figure 1. Upon ligation with T4 RNA ligase 2 (lane 6) additional bands appear (Band III - V) likely corresponds to large ligation products (the RNA multimer complexes) that are now ligated together and thus stable in the denaturing gel. Interestingly, the 33 nt circles, representing a whole integer of the helical pitch (11 bp/turn), leads to much stronger ligation products compared to when the 36 nt circle, which is not a whole integer of the helical pitch.


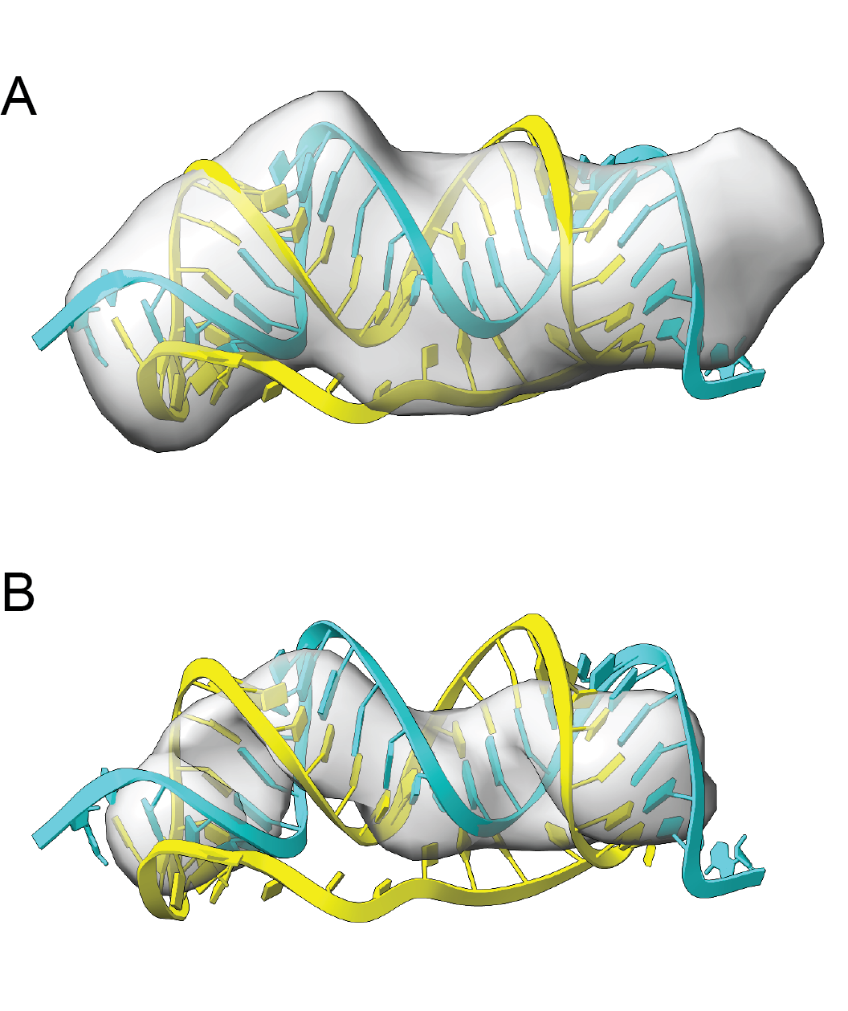


# Figure S11. Fitting of MD model to class 1 density.

Fitting of previously modelled RNA system (in main text ref.: 29) (consisting of 36 nt scRNA and 30 nt cmpRNA) into class 1 density. (A) shows the density at a cut-off value of 0.35 where the density mostly covers the model. (B) shows the density at a cut-off value 0.75 to show the helical feature of the density.


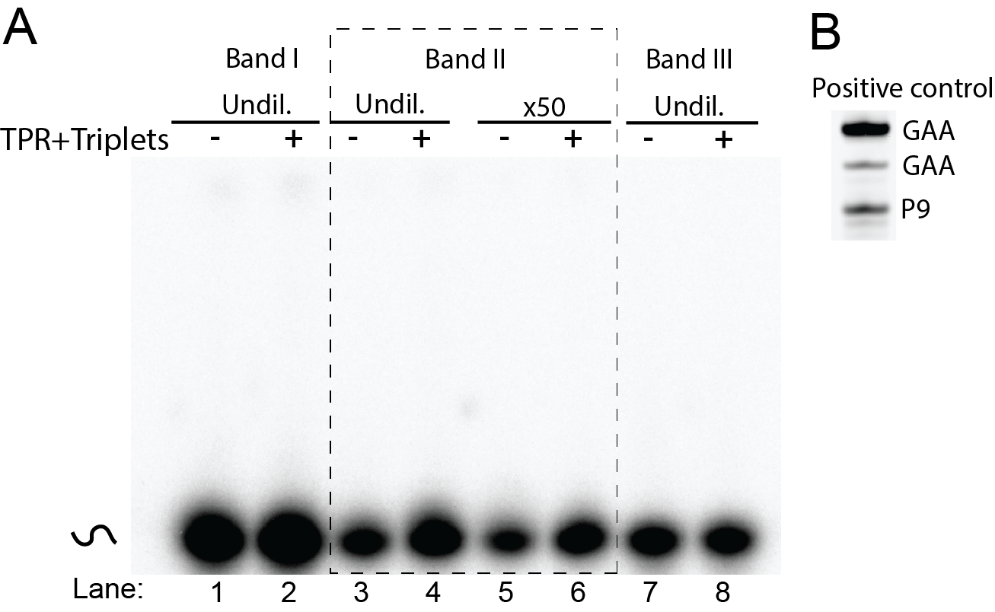


# Figure S12. Primer extension scRNA species by TPR.

Denaturing gel (20% 19:1) of gel-purified RNA after incubation with active TPR and reaction components. (A) All three bands (I-III) observed in the native gel in main text Figure 1C was gel-purified and the products incubated with active TPR and triplets to extend the hot-labeled cmpRNA. None of the gel purified products (the single stranded cmpRNA without template (band I, lane 1 and 2), the homodimer (band II, lane 3-6) or the multimer (band III, lane 7 and 8)) lead to extension of the cmpRNA during the reaction. x50 in lane 5 and 6 denotes that the sample had been 50-fold diluted prior to incubation, which had previously been shown to improve the RCS reaction. This did not work either. (B) Positive control showed efficient primer extension by TPR confirming that the reaction components were active.


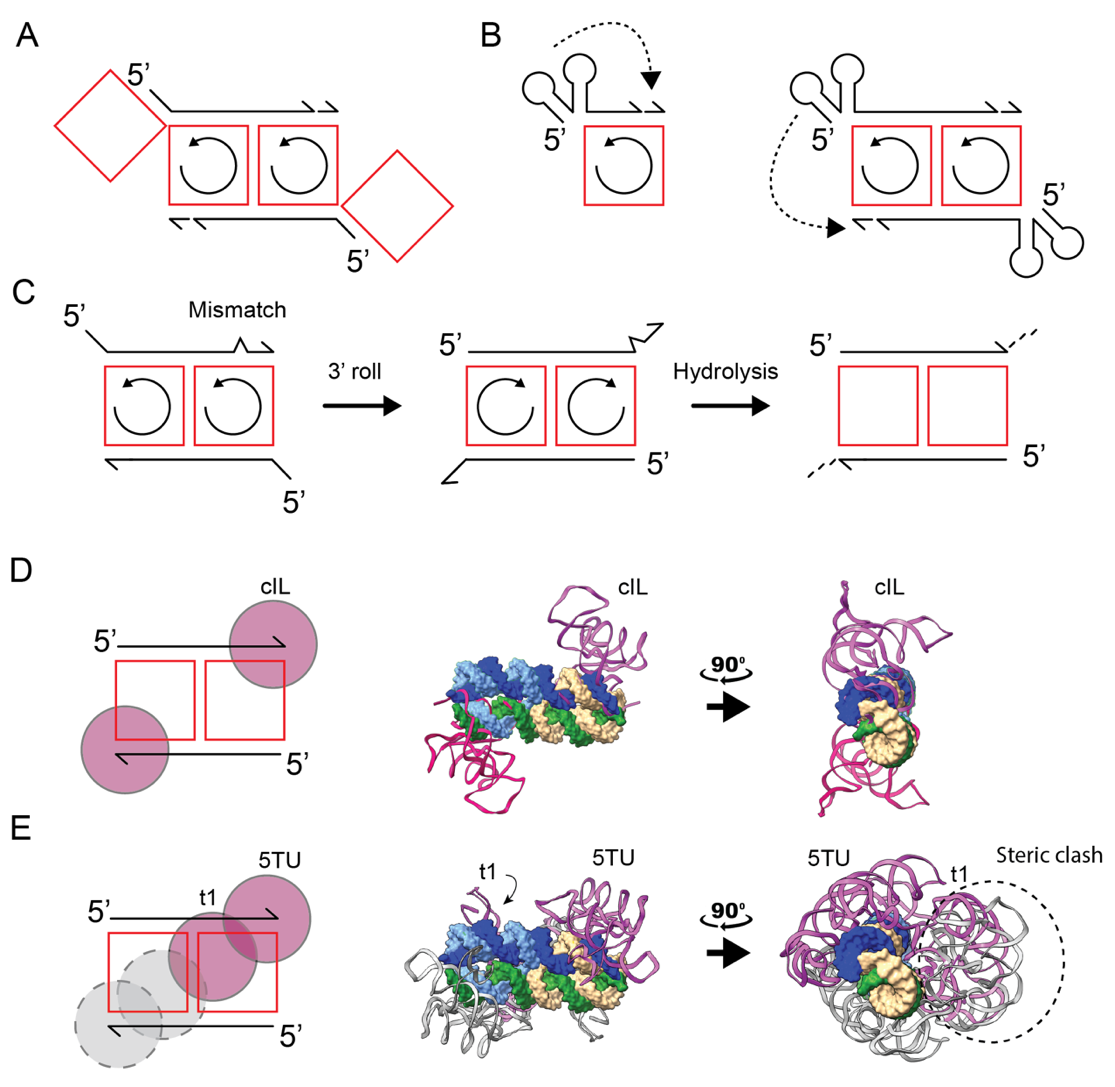


# Figure S13. Dimeric rolling circle replication hypothesis.

(A) Schematics showing how additional circular templates may bind to 5’-end and drive directionality of synthesis. (B) Schematics of monomer and dimer RCS, where secondary structure formation drives directionality of synthesis. Dashed line with arrow indicates how a nascently folded RNA polymerase ribozyme may act within the template-product complex. (C) Schematics showing how a mismatch may induce 3' rolling and how the exposed strand may hydrolyze to reset the synthesis.

Movie 1. Illustrative movie of the rolling circle assembly mechanism based on the observed structural classes.

Movie 2. Illustrative movie of the hypothesized dimeric rolling circle polymerization mechanism where each 3'-end is continuously extended which in turn causes concerted 5'-end extrusion.
